# A protection strategy for distribution networks using IoT technology and support vector machine

**DOI:** 10.1038/s41598-026-66687-8

**Published:** 2026-08-26

**Authors:** Esraa M. Shalby, Almoataz Y. Abdelaziz, Eman S. Ahmed, Basem Abd-Elhamed Rashad

**Affiliations:** 1 Faculty of Engineering, Ain Shams University, Cairo, 11517 Egypt; 2 Department of Electrical Power and Machines Engineering, Higher Institute of Engineering at El-Shorouk City, El-Shorouk Academy, Cairo, 11837 Egypt; 3 Faculty of Engineering and Technology, Future University in Egypt, Cairo, 11835 Egypt; 4 Department of Electrical Engineering, Faculty of Engineering, Kafrelsheikh University, Kafrelsheikh, Egypt

**Keywords:** Electrical power system protection, IoT, MATLAB classification learner app (MCLA), MATLAB regression learner app (MRLA), Support vector machine, ThingSpeak, Energy science and technology, Engineering, Mathematics and computing

## Abstract

This paper presents an intelligent protection framework for fault detection, classification, and location in power distribution networks by combining Discrete Wavelet Transform (DWT)-based feature extraction, Support Vector Machine (SVM)-based decision making, and Internet of Things (IoT)-enabled cloud monitoring. An IEEE 16-bus distribution system is modeled in MATLAB/Simulink, where transient current signals are processed using DWT to extract discriminative time–frequency features. A comparative evaluation of different mother wavelets and decomposition levels is performed to identify the most effective feature extraction configuration in terms of accuracy and computational efficiency. The extracted features are processed locally by SVM-based models for fault detection, classification, and location, while selected fault-related features are simultaneously transmitted to the ThingSpeak cloud platform for cloud-assisted monitoring and remote accessibility. The proposed framework is evaluated under a wide range of operating conditions, including different fault types, overload events, load switching, capacitor switching, and scenarios with integrated photovoltaic and wind generation. The results demonstrate 100% fault classification accuracy and fault-location accuracies ranging from 97.95% to 99.88% within the investigated simulation scenarios. Furthermore, the proposed approach effectively distinguishes faults from non-fault disturbances, thereby reducing the likelihood of false fault indications. The findings demonstrate that the proposed intelligent protection framework provides accurate and reliable fault detection, classification, and location through optimized DWT-based feature extraction and SVM-based decision making under the investigated simulation scenarios. Nevertheless, additional validation using noisy measurements and hardware-based experimental platforms is required to further assess the robustness and practical applicability of the proposed protection methodology under real operating conditions.

## Introduction

### Paper motivation

In modern electrical distribution systems, fault protection is essential for ensuring uninterrupted power supply. Integrating the Internet of Things (IoT) and Machine Learning (ML) enables autonomous, self-healing networks. Intelligent devices and distributed control algorithms detect and isolate faults, reconfigure topology, and restore service in real time^1^. IoT provides a multidisciplinary framework with applications in environmental, industrial, medical, and transportation sectors, each tailored to specific research domains^2^. Kevin Ashton coined the term “Internet of Things” (IoT) in 1999^3^, and it remains central to today’s technological advancements. An IoT-based smart net-metering system, developed in MATLAB/Simulink, demonstrates real-time monitoring and control for bidirectional energy flow^4^, while IoT integration enhances the efficiency and reliability of electric vehicle supercapacitor systems^5^. Cloud-based IoT monitoring improves inspection accuracy and efficiency^6^, and IoT sensors ensure environmental safety by tracking air quality affected by nuclear activities^7^. Additionally, IoT supports remote transformer fault monitoring^8^, and provides cloud-based fault diagnosis solutions within electrical distribution networks^9^.

Power grids have evolved rapidly and are crucial to national economic growth. Efficient generation, transmission, and distribution are vital, with the distribution network serving as the final link delivering energy to consumers. Rising energy demand has expanded feeders and distribution lines to meet population needs^10^. Faults in distribution systems often arise from short circuits or environmental disturbances. While temporary faults may self-clear, neglect can escalate them into permanent ones due to insulation failures or mechanical impact^11^. Fault management faces challenges in distinguishing between fault types, differentiating temporary from permanent faults, and precisely locating them. These complexities exceed conventional protection capabilities, emphasizing the need for adaptive solutions based on Artificial Intelligence (AI). AI-based fault identification techniques and their advantages are discussed in^12^, while^13^ explores the strengths, challenges, and trends of Support Vector Machines (SVMs). Comprehensive studies on IoT-enabled smart grids address key technologies, architectures, and challenges^14^, including the integration of IoT and digital twin technologies in power networks^15^.

### Literature review

Accurate fault detection, classification, and location are critical for reliability. Integrating Wavelet Packet Transform (WPT) and SVM enhances fault detection accuracy to 98.56%^16^. Techniques combining Support Vector Regression (SVR), Relevance Vector Machine (RVM), and Stationary Wavelet Transform (SWT) have also been proposed^17^. Wavelet Packet Decomposition (WPD) with SVM further improves classification accuracy^17^, and a WPT–SVM algorithm achieved 99% accuracy on the Eskom 90-bus system^18^. SWT combined with SVM, Artificial Neural Network (ANN), and SVR improves diagnostic accuracy^19^. SVMs with Radial Basis Function (RBF) kernels achieved high accuracy on IEEE 34- and 123-bus systems^20^, while Directed Acyclic Graph-SVM (DAG-SVM) effectively classified faults based on voltage sag magnitudes^21^. Other approaches used K-Nearest Neighbors (KNN), Decision Tree (DT), and SVM with wavelet decomposition, attaining high accuracy on the IEEE 33-bus system^22^. ML-based fault classification under different fault resistances for IEEE 16-bus systems is presented in^23^. A DWT–SVM technique utilizing single-bus measurements demonstrated strong performance^24^. Similarly, Karrenbauer Transform and SVR achieved 98.56% accuracy^25^. Methods using SVMs to identify fault type, phase, and location achieved satisfactory accuracy^26^, while DWT–SVM approaches for IEEE 33-bus systems provided precise fault detection and self-healing capabilities^27^. Smart meter–based SVMs also achieved fault location accuracy exceeding 87%^28^.

A major challenge in distribution protection lies in differentiating normal conditions, transient disturbances, and genuine faults^29^. Traditional systems often misinterpret short events such as capacitor switching or load changes, resulting in false trips or missed detections^29^. These inaccuracies reduce reliability and raise maintenance costs. The overlapping signatures of transient and fault signals are the core issue. ML-based techniques, in contrast, offer faster, adaptive, and intelligent solutions but remain underutilized, especially in real-time applications. A key research gap is the integration of real-time IoT data streams with ML models for immediate fault detection and adaptive protection^30^. Selecting the optimal ML technique for tasks such as identifying, classifying, and locating faults remains a challenge^23^. Many studies rely on limited evaluation metrics, resulting in suboptimal performance under diverse conditions.

**Table 1:** Comparison between traditional fault detection methods and machine learning-based approaches for distribution system protection.

Aspect	Traditional fault detection methods	Machine learning-based approaches
Detection principle	Threshold-based techniques (e.g., overcurrent and impedance-based protection)	Data-driven techniques (e.g., SVM, Naïve Bayes, Artificial Neural Networks)
Capability for complex faults	Limited capability in detecting high-impedance and evolving faults	Effective in identifying various fault conditions, including complex and high-impedance faults
Adaptability to network changes	Requires manual setting adjustments and periodic coordination	Learns from historical data and can be retrained to accommodate changing operating conditions
Performance under DG penetration	May experience degraded performance due to altered fault characteristics	Better suited to capturing nonlinear relationships introduced by distributed generation
Detection accuracy	Dependent on relay settings and system conditions	Typically achieves higher accuracy when appropriate feature extraction and training datasets are employed
Computational requirements	Generally low	Moderate, depending on the selected learning algorithm
Real-time implementation	Mature and widely deployed but less flexible	Fast decision-making capability with suitable feature extraction and optimized classifiers
Dependence on system models	Strong dependence on network parameters and predefined assumptions	Reduced dependence on explicit mathematical models through data-driven learning

The integration of Distributed Energy Resources (DERs) has enhanced network reliability and voltage profiles but also increased system complexity^31^. Fault current magnitude depends on DER type, and the inclusion of Distributed Generators (DGs) can impair relay performance in detecting and classifying faults^32^. In photovoltaic (PV) systems, the Perturb and Observe (P&O) method remains standard for maximum power point tracking (MPPT)^33^. Addressing these challenges requires hybrid frameworks combining robust signal processing and ML algorithms for accurate fault classification and location. Although power system protection has advanced, notable gaps persist. Traditional protection relies on fixed thresholds and relays, while AI-based systems offer dynamic, data-driven adaptability. Machine learning classifiers such as SVMs, KNNs, and DTs can extract unique fault features from transient signals. Table 1 compares conventional and ML-based protection approaches, highlighting the shift toward intelligent fault management^30,34^.

A summary of recent wavelet-based machine learning approaches is presented in Table 2, highlighting variations in network models, targeted protection tasks, consideration of non-fault disturbances, integration of distributed generation, and real-time or IoT-enabled implementation. This comparison illustrates that, although DWT- and ML-based methods remain prevalent in contemporary studies, most existing approaches address only specific aspects of the protection problem. In contrast, the proposed framework provides a comprehensive solution for fault detection, classification, and location, validated under both faulted and non-faulted conditions and accommodating the presence of renewable energy sources. Table 3 presents a comparative analysis between the Discrete Wavelet Transform (DWT) and alternative signal processing techniques to justify the selection of DWT from a protection-oriented perspective. The comparison demonstrates that DWT provides an optimal balance between time–frequency resolution, sensitivity to transient disturbances, computational efficiency, and feasibility for real-time implementation. These characteristics make DWT a robust and widely recognized technique for fault detection and protection applications in modern distribution networks.

**Table 2:** Summary of related works-based wavelet analysis with AI techniques.

Ref.	Year	Signal processing	Learning model	Network type	Disturbances	DG integration	Real-time suitability	Classification accuracy
R^35^	2021	WPT	SVM	Transmission line	LIFs	No	Moderate	98.85%
R^36^	2021	DWT	SVM	IEEE 33-bus	LIFs	No	High	99.03%
R^27^	2022	DWT	SVM	IEEE 33-bus	LIFs	No	High	100%
R^37^	2023	CNN	SVM	IEEE 123-bus	LIFs	No	Moderate	98.36%
R^38^	2023	DWT	SVM	IEEE 9-bus	LIFs	No	High	99.30%
R^39^	2024	DWT	DNN	Two Area cluster	LIFs	Yes	Moderate	100%
R^40^	2024	DWT	*RBFNN*	IEEE-5 bus	LIFs	Yes	Moderate	100%
R^41^	2025	DWT	SVM	IEEE 16-bus	LIFs	Yes	High	100%
R^42^	2025	-	SVM	IEEE 34-bus	LIFs	No	High	100%
This work	2026	DWT	SVM	IEEE 16-bus	Faults + Switching + Overloads	Yes	High	100%

CNN= Convolutional Neural Network; DNN= Deep Neural Network; DWT= Discrete Wavelet Transform; RBFNN=Radial Basis Function Neural Network; WPT=Wavelet Packet Transform.

**Table 3:** Comparison between DWT and other signal processing techniques.

Evaluation aspect	Wavelet-based analysis (DWT)	Fourier-based analysis (FT / STFT)	Adaptive decomposition (HHT)	Learning-driven approaches (ML/DL)	References (2022–2026)
Spectral–temporal representation	Multi-scale time–frequency representation with adaptive resolution	Frequency-focused or window-limited time–frequency mapping	Data-adaptive instantaneous frequency representation	Implicit feature representation learned from data	R^43^, R^44^
Suitability for non-stationary events	Highly effective for transient and abrupt disturbances	Limited, especially for rapidly varying signals	Strong capability for non-linear and non-stationary signals	Strong if trained on representative non-stationary patterns	R^45^
Computational burden	Low to moderate; suitable for embedded platforms	Low (FT), moderate (STFT)	High due to iterative decomposition	High during training; moderate during inference	R^43^, R^46^
Dependence on prior data	Minimal (signal-driven)	Minimal	Moderate (sensitive to preprocessing)	Significant (requires labeled datasets)	R^47^
Physical interpretability	High-clear correspondence between coefficients and signal features	High in frequency domain; limited temporal insight	Moderate; interpretation may be mode-dependent	Low-decision process is often opaque	R^48^
Real-time protection feasibility	Well-suited for fast protection schemes	Generally unsuitable without window compromise	Typically, unsuitable for strict real-time constraints	Application-dependent; latency tied to model complexity	R^49^
Scalability to multiple fault scenarios	Effective across diverse fault types	Limited discrimination capability	Effective for dynamic signal variations	Highly scalable if trained on diverse fault classes	R^41^, R^50^
Hybrid integration potential	Commonly used with ML classifiers	Rarely used in hybrid frameworks	Occasionally combined with ML	Naturally supports hybrid and ensemble designs	R^51^, R^52^
Deployment maturity in protection systems	Widely adopted in practical and academic studies	Mostly analytical or educational use	Primarily research-oriented	Rapidly emerging; limited field deployment	R^53^

Table 4 summarizes AI-based fault detection and location techniques applied to IEEE distribution systems. Early works^54,55^ using ANN and SVM achieved high classification accuracy but lacked communication integration. Later studies^27,37^ introduced CNN-based feature extraction, improving accuracy and scalability but not addressing DG or transient conditions. LSTM–CNN hybrid models in^56^ achieved strong performance with DGs but omitted communication aspects. Recent contributions^23,41^ integrated DWT-based feature extraction with advanced classifiers such as Gaussian Naïve Bayes and SVM, achieving 100% accuracy on the IEEE 16-bus system. The proposed method advances this field by combining IoT-based communication, DWT-based feature extraction, and hybrid ML classifiers, achieving 100% fault detection and classification accuracy and near-perfect fault location accuracy, while effectively distinguishing transient disturbances such as load and capacitor switching.

**Table 4:** A brief of the literature associated with classifying and locating faults in electrical power networks using AI- techniques.

Ref.	*R* ^54^	*R* ^55^	*R* ^27^	*R* ^37^	*R* ^56^	*R* ^23^	*R* ^41^	Proposed technique
Year	2020	2022	2022	2023	2024	2024	2025	2026
Study system	IEEE-16 bus	11-node IEEE	IEEE 33-bus	IEEE 123-bus	IEEE 9-bus System	IEEE-16 bus	IEEE-16 bus	IEEE-16 bus
Communication technique	–	–	–	–	–	–	–	IoT
Feature extraction	–	Fast Fourier Transform (FFT)	DWT	CNN	CNN	DWT	DWT	DWT
AI-technique	ANN	SVM	SVM	SVM	LSTM	Gaussian Naïve Bayes	SVM	Hybrid ML techniques
Accuracy of fault classification	99.90%	94.24%: 97.87%	–	98.36%:100%	–	100%	100%	100%
Accuracy of fault classification with DGs	99.73%: 99.97%	–	100%	–	99.52%: 100%	–	100%	100%
Accuracy of fault location	92.14%: 97.04%	–	–	–	–	–	–	97.95%: 99.88%
Accuracy of fault location with DGs	81.90%: 93.37%	–	–	–	99.10%: 99.63%	–	–	97.87%: 98.45%
Load/capacitor switching	–	–	–	–	–	–	✓	✓
Overload	–	–	–	–	–	–	–	✓

### Paper contribution and organization

The scientific contribution of this work does not arise from the introduction of new machine-learning algorithms or signal-processing techniques, since both DWT and SVM are well-established methodologies. Instead, the contribution lies in the development of an integrated intelligent protection framework that combines optimized DWT-based feature engineering, SVM-based fault detection, classification, and location, and cloud-assisted monitoring within a unified protection architecture. The framework is further strengthened through the systematic evaluation of wavelet functions, decomposition levels, machine-learning models, fault scenarios, non-fault disturbances, and renewable-integrated operating conditions, while explicitly distinguishing the real-time protection decision path from the cloud-monitoring function.

Main contributions and innovations: (i) Comprehensive fault analysis of the IEEE 16-bus distribution system under symmetrical faults, asymmetrical faults, non-fault disturbances, and renewable-integrated operating conditions. (ii) Validation of the proposed framework under renewable-integrated operating conditions through the incorporation of 9 MW photovoltaic and 9 MW wind generation models, demonstrating the applicability of the proposed protection framework in active distribution networks with high distributed generation penetration. (iii) Systematic optimization of DWT feature extraction through comparative evaluation of multiple wavelet families and decomposition levels, leading to the selection of the Sym3 wavelet at decomposition level 8 based on both classification performance and computational efficiency. (iv) Development of an integrated intelligent protection framework that combines real-time current acquisition, optimized DWT feature engineering, SVM-based fault detection, classification, and location, together with cloud-assisted monitoring in a unified protection architecture. (v) Comprehensive evaluation of fourteen machine-learning classifiers and multiple SVM regression models to identify the most suitable models for classification accuracy, prediction speed, computational efficiency, and fault-location performance. (vi) Comprehensive validation of the proposed framework under diverse operating scenarios, including symmetrical and asymmetrical faults, overload conditions, load switching, capacitor switching, and renewable-integrated operating conditions, demonstrating its robustness and reliable performance under the investigated simulation scenarios. (vii) Explicit separation of the local protection decision path from the cloud-monitoring function, thereby clarifying the operational role of the IoT platform within the proposed architecture. (viii) Achievement of 100% fault-classification accuracy and 97.95–99.88% fault-location accuracy under the investigated simulation scenarios. (ix) Clearly defining the scope of the present study by identifying hardware validation, communication latency, measurement uncertainty, and advanced inverter dynamics as important directions for future research and practical implementation.

This paper presents a novel technique for fault detection, classification, and location in electrical distribution systems by integrating the Internet of Things (IoT), Discrete Wavelet Transform (DWT), and Support Vector Machine (SVM). The proposed method, implemented on the IEEE 16-bus system, achieves 100% fault classification accuracy and fault location accuracy above 97% with and without distributed generations (DGs), demonstrating high reliability and robustness. Section  2 details the proposed scheme, including DWT-based feature extraction, IoT integration, and SVM implementation using MCLA and MRLA. Section  3 explains the Discrete Wavelet Transform (DWT) and its challenges. Section  4 introduces ThingSpeak, a cloud-based IoT platform. Section  5 discusses Support Vector Machines (SVMs), including kernel types and classification and regression methods. Section  6 describes the modeling of renewable energy sources. Section  7 describes the IEEE 16-bus test system. Section  8 presents results under different fault conditions, and Sect.  9 describes the practical considerations and limitations. Section  10 concludes the study, confirming the model’s accuracy and effectiveness for modern distribution networks.

## Proposed fault detection, classification and location scheme

**Fig. 1:**
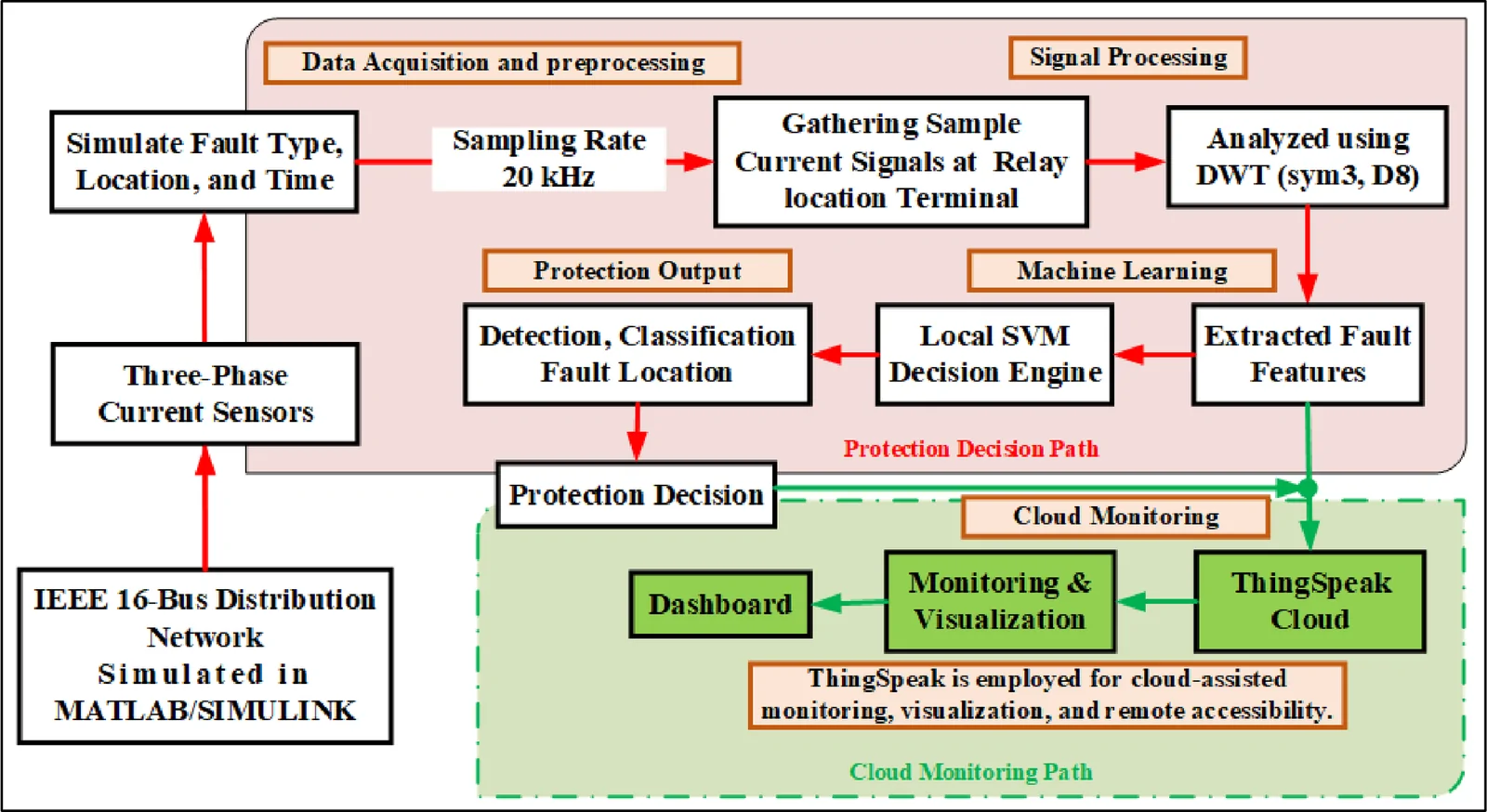
The complete signal-processing and decision-making architecture.

Figure 1 illustrates the overall architecture of the proposed intelligent protection framework, which integrates signal acquisition, signal processing, local machine-learning-based decision making, and cloud-assisted monitoring for fault detection, classification, and location in the IEEE 16-bus distribution network. The architecture is organized into six sequential processing stages, while clearly distinguishing the real-time protection decision path from the independent cloud-monitoring path. For clarity, the six processing stages are grouped into four functional modules: (i) data acquisition and preprocessing, (ii) DWT-based signal processing and feature extraction, (iii) local SVM-based protection decision making, and (iv) cloud-assisted monitoring. **Step 1 (Data Acquisition and Preprocessing)**: Three-phase current signals are acquired from strategically positioned current sensors installed at electrically significant locations throughout the IEEE 16-bus distribution network. The sensors continuously monitor the network currents and provide continuously sampled three-phase current measurements at 20 kHz, corresponding to 400 samples per fundamental cycle (50 Hz). Following fault inception, a one-cycle post-event current window is extracted and forwarded to the signal-processing stage.

**Step 2 (Signal Processing using DWT)**: The acquired current signals are processed using the Discrete Wavelet Transform (DWT) implemented with the Symlet 3 (sym3) mother wavelet at decomposition level 8, as identified through the comparative analysis presented in this work. The DWT isolates the transient fault components in both the time and frequency domains, enabling reliable extraction of fault-sensitive information. **Step 3 (Feature Extraction)**: The maximum value of the level-8 detail coefficient (D8) is extracted from each analyzed signal to form the feature vector supplied directly to the local SVM-based decision engine. These features effectively characterize transient fault behaviour while maintaining a low computational burden suitable for intelligent protection applications. **Step 4 (Machine-Learning Decision Engine)**: The extracted DWT features are processed locally by the trained Support Vector Machine (SVM) models to perform fault detection, fault classification, and fault location. The classification models were developed using the MATLAB Classification Learner App (MCLA), whereas the fault-location models were implemented using the MATLAB Regression Learner App (MRLA). The optimal models were selected based on classification accuracy, prediction speed, training time, and Root Mean Square Error (RMSE). **Step 5 (Protection Decision)**: Based on the outputs of the SVM decision engine, the proposed protection framework generates the corresponding protection decision by identifying the fault type and estimating its location. This decision-making process is executed entirely within the local protection framework and is independent of any cloud communication. **Step 6 (Cloud-Assisted Monitoring)**: In parallel with the local protection process, the selected DWT fault features are transmitted to the ThingSpeak cloud platform exclusively for remote monitoring, visualization, and data accessibility. The cloud platform is not involved in the protection tripping path and does not participate in the real-time fault detection, classification, or location process. Accordingly, temporary communication delays or loss of cloud connectivity do not affect the execution of the local protection algorithm. Consequently, the operation of the proposed protection framework remains independent of Internet connectivity and cloud communication latency, such that protection decisions are executed exclusively within the local processing framework.

## Discrete wavelet transform (DWT)

DWT is a specific implementation of the wavelet transform, discretizing both the wavelet functions and the signal for effective analysis in discrete time and space. DWT finds extensive use in various applications like data analysis, image compression, and signal processing. Rooted in mathematics, wavelet theory consolidates small signals into a singular one, represented by a wavelet possessing distinct wave-like characteristics. These characteristics involve oscillation starting at zero, rising in amplitude, and returning to zero. Signals within wavelet theory are compositions of wavelet clusters derived from the same mother wavelet through alterations in size (scaling: m) or position (shifting k) along the time axis^57^. The signal analysis facilitated by DWT permits the selection of various positions (k) and scales (m) to combine wavelets, enabling the creation of a signal at a particular scale of interest. Through integrating the signal across all resolutions, the original input signal is reconstructed. In hierarchical analysis, each iteration progressively halves the scale of examination. Therefore, this process exemplifies a DWT-type discrete wavelet transform, as depicted in Eq. (1)^57^.

1 $$\:DWT(m,n)=\frac{1}{\sqrt{{2}^{m}}}\sum\:_{k}f\left(k\right)\psi\:[\frac{n-k{2}^{m}}{{2}^{m}}]$$

where: $\:\psi\:=Mother\:wavelet$, * m*, * n*, * k* are Integers, * m* = Scaling, * k* = Shifting * n* = Number of data points.

In Discrete Wavelet Transform (DWT), a signal is progressively broken into detail and approximation coefficients using successive high-pass and low-pass filters, forming the analysis filter bank. Detail coefficients capture fine variations, while approximation coefficients reflect broader patterns. Repeated decomposition of the approximation coefficients enables multiresolution analysis. The frequency divisions at each level follow the Mallat algorithm and adhere to Nyquist’s theorem. As principle of Nyquist, the maximum resolvable frequency equals half the sampling rate, meaning a signal sampled at F Hz has a maximum frequency of F/2 Hz^58^.

In this study, the power system operates at a fundamental frequency of 50 Hz, and three-phase current signals are sampled at a rate of 20 kHz. Consequently, one fundamental cycle corresponds to 20 ms, yielding 400 samples per cycle. Following fault inception, a post-event data window of one fundamental cycle is extracted and used for feature computation. This window length provides a suitable balance between capturing the essential high-frequency transient characteristics associated with fault conditions and maintaining low computational complexity. The selected post-event window is processed using the Discrete Wavelet Transform to extract time–frequency features, which are subsequently normalized and fed to the SVM-based classifier for fault detection, classification, and location. The use of a single-cycle post-event window supports rapid and reliable protection decisions, making the proposed approach well suited for real-time implementation in distribution networks.

### Decomposition level selection

**Fig. 2:**
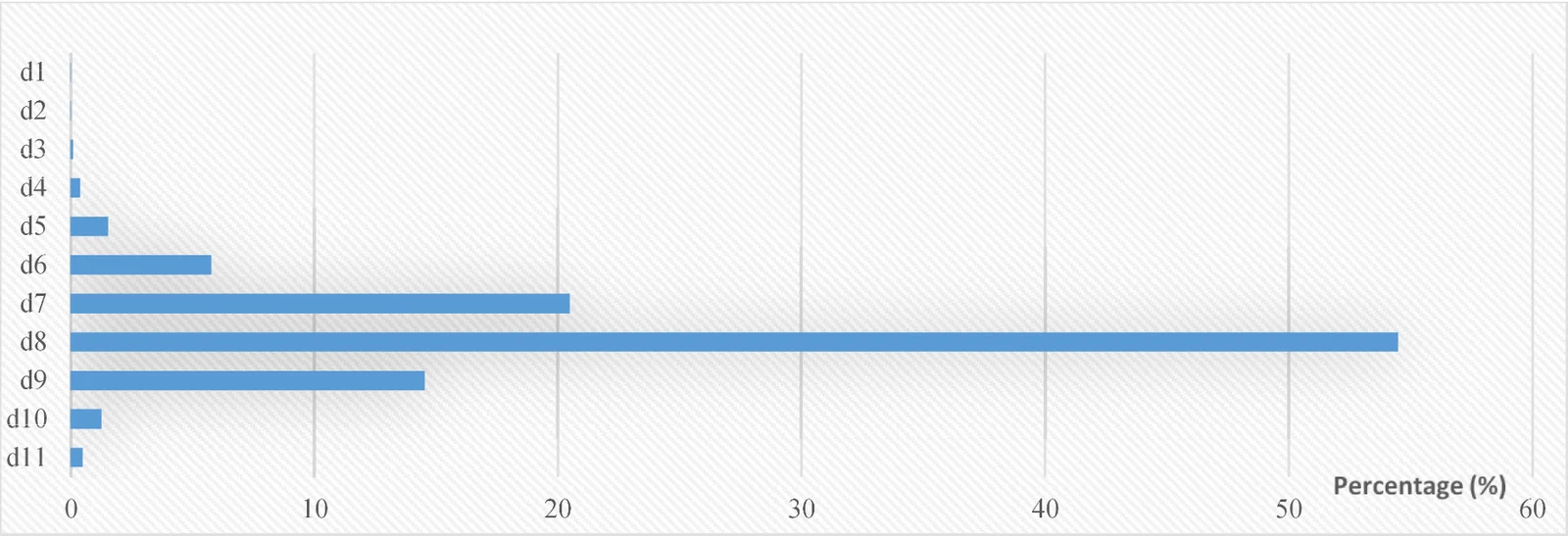
The highest percentage of energy transient current signal is d8.

**Fig. 3:**
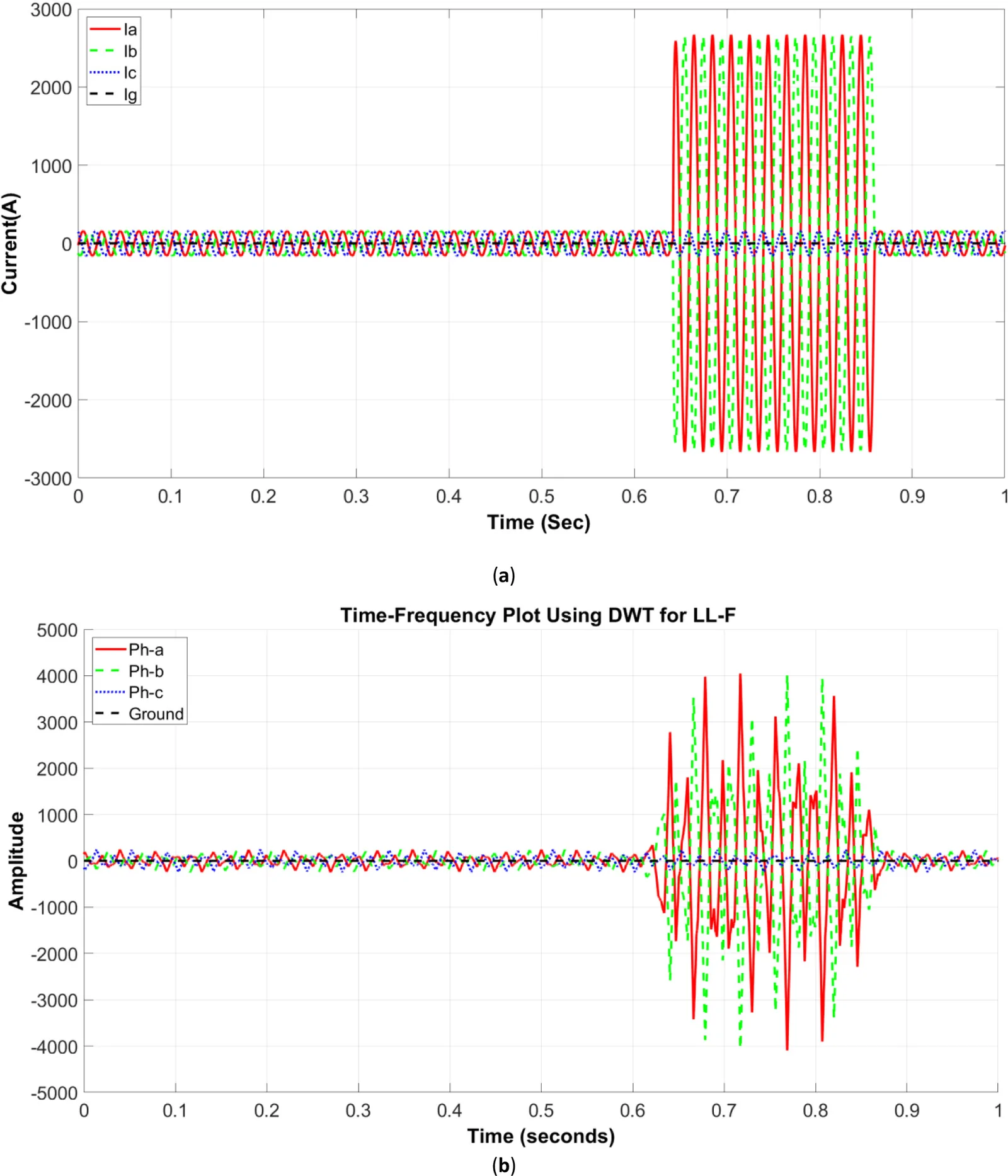
The initial transient current waveform and its corresponding decompositions for B-C fault at 45˚ fault inception angle. (**a**) Three phase current waveform. (**b**) Time-frequency response using DWT at Level 8.

MATLAB Wavelet Toolbox is employed to assess the current signal and determine the appropriate wavelet level. As shown in Fig. 2, which indicates the highest percentage of transient current energy is at (d8)^41^, and The initial transient current waveform and its corresponding decompositions for B-C fault at 45° fault inception angle is illustrated in Fig. 3.

### Suitable wavelet function and feature extraction

In Wavelet Transform (WT) analysis, selecting an appropriate mother wavelet is crucial due to its shape variations designed for specific applications. The main challenge is identifying the optimal wavelet to achieve accurate results and a strong correlation with the signal. In^59^ outline key properties to consider when selecting wavelet functions, with a summary of popular wavelet functions. While^41^ explore different wavelet families, such as Daubechies, Biorthogonal, Meyer, Symlets, Reverse BiorsSplines, and Coiflet wavelets. Selecting the appropriate mother wavelet is indeed a critical decision in applications like fault detection, as it can significantly influence the classification training time, prediction speed and accuracy of the model. MATLAB Wavelet Toolbox provides various wavelet functions that can be tailored to optimize these factors. While db5 proves effective, **sym3** achieves higher classification accuracy, particularly in fault detection^41^. For this application, **sym3** is therefore the recommended mother wavelet. The key step involves extracting the effective important feature from the **sym3** wavelet at the eighth level (**d8**), this maximum feature captures critical characteristics of the fault current and then sends it to the cloud.

## ThingSpeak: cloud-based IOT platform

**Fig. 4:**
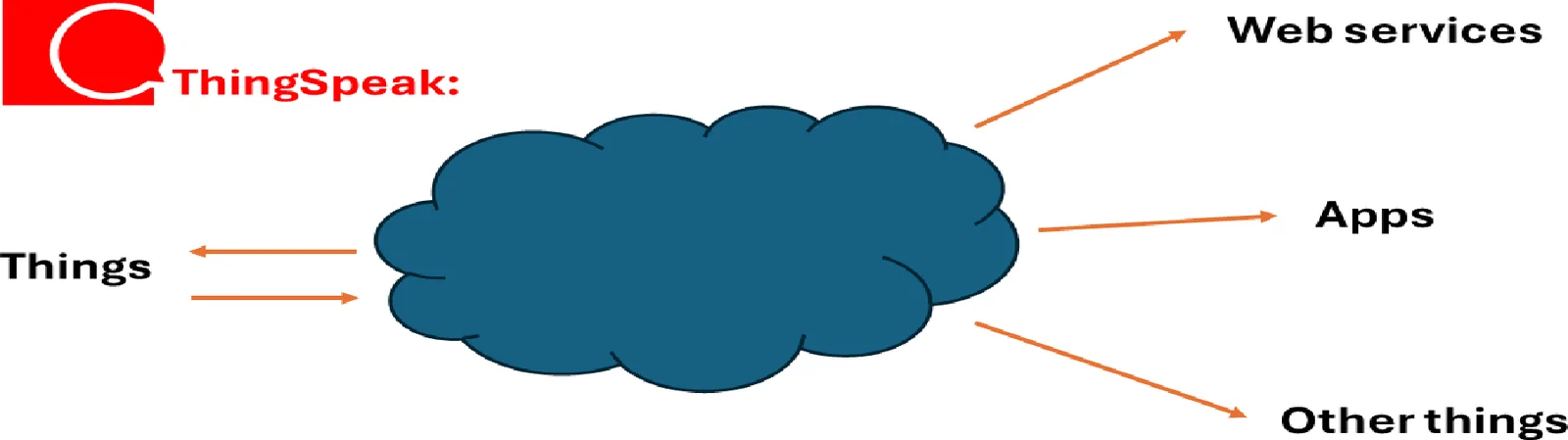
ThingSpeak represented as ‘Cloud’ interface.

ThingSpeak is an online open Application Programming Interface (API) specifically developed for Internet of Things (IoT) applications, known for its extensive capabilities in storing sensor data from diverse applications. The functionality of its API is comprehensively outlined on its website^60^ as shown in Fig. 4. The platform facilitates the visualization of sensor data through graphical representations on the web. Central to ThingSpeak’s functionality is its ‘Channels’ concept, which features fields for data, location, and status, accommodating various types of sensor data. Once channels are configured within ThingSpeak, users are able to view and analyze real-time data^61^. Additionally, ThingSpeak supports the creation of public channels, which can be utilized for collaborative analysis and estimation. The ‘Cloud’ feature of ThingSpeak operates through graphical visualization and is accessible via virtual servers^62^. Communication with the cloud is conducted through available wireless internet connections, with many objects utilizing sensors and actuators to transmit analog environmental data. IoT is essential in unifying these elements, enabling communication between users and their devices, and facilitating interactions among various ‘things’^62^.

## Support vector machines (SVMs)

SVMs is a popular supervised learning technique introduced by Cortes and Vapnik in 1995 and is a versatile tool for both regression and classification tasks^63^. SVM is referred to as SVC when applied to classification tasks^64^. SVM is known as SVR when utilized for solving regression problems^65^. SVC is used to identify fault types, whereas SVR is applied to determine the location of faults^66^. SVM model is beneficial as it avoids local minima, resists overfitting, maintains sparsity, and provides global solutions^67^. SVMs create a hyperplane that optimally separates classes by maximizing the margin from the closest data points, defined by a dot product with a vector in higher-dimensional space, and uses linear combinations of feature vectors to handle both linear and nonlinear patterns while minimizing generalization^68^. The nearest data points to the hyperplane, called Support Vectors (SVs), determine the decision boundary^69^. Since imperfect decision boundaries can lead to misclassification, these critical data points help define the limits while disregarding other training samples^69^.

### Support vector machines for classification (SVC)

**Fig. 5:**
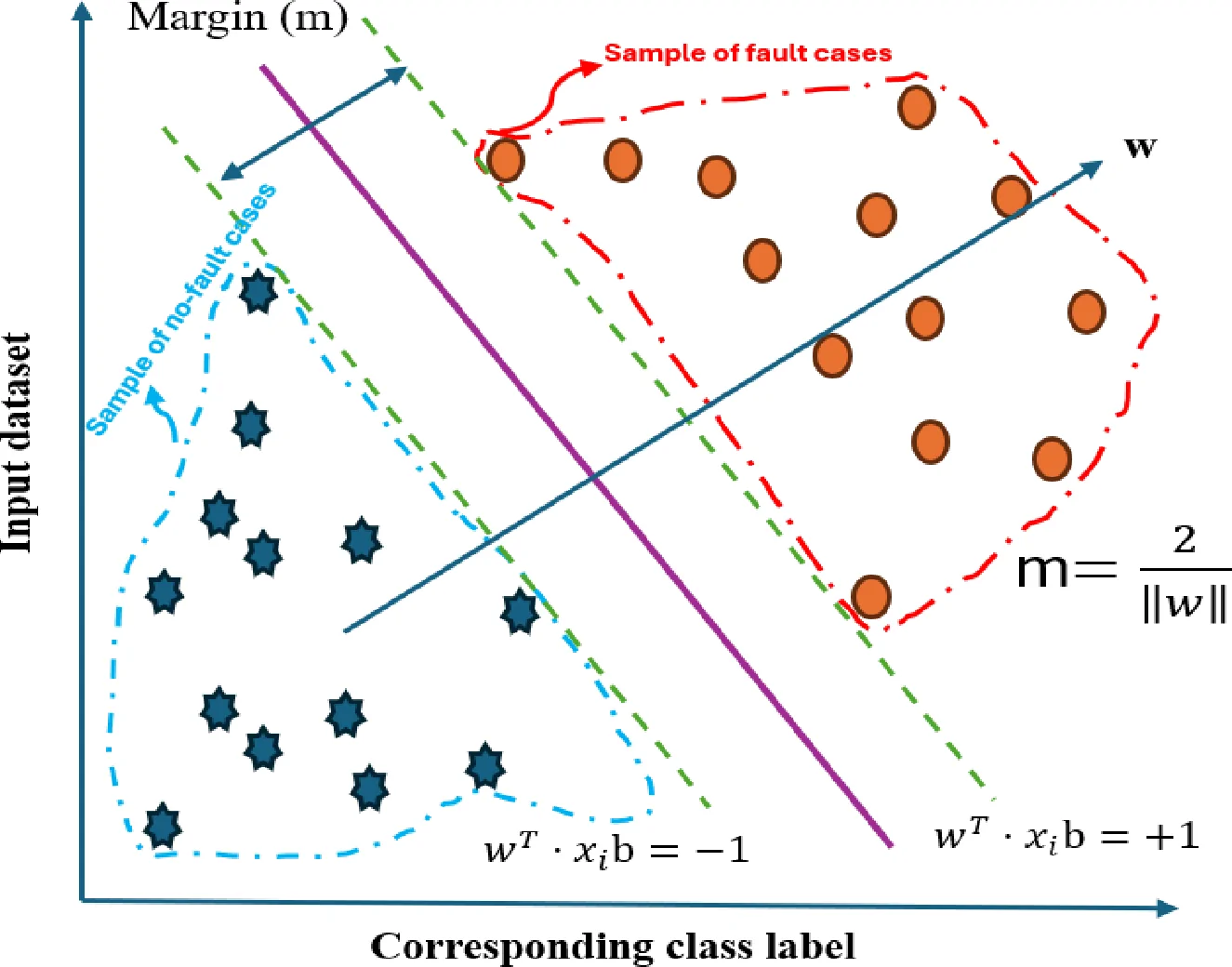
Hyper-plane of SVM classifier.

SVC is supervised models that employ algorithms to examine data, recognize patterns, and determine which of two possible classes an input belongs to, operating as a non-probabilistic binary linear classifier^70^. SVC performs exceptionally well on unseen data by leveraging its fundamental classification principle to correctly assign new instances to the right category^68^. Consider a dataset consisting of two classes of samples, where each sample is represented by $\:{x}_{i}\:$ and its corresponding class label $\:{y}_{i}$: $\:{\mathrm{x}}_{\mathrm{i}}$∈ $\:{\mathrm{R}}^{\mathrm{n}}$, $\:{\mathrm{y}}_{\mathrm{i}}\:$ ∈ {-1,1}, i = 1,2,3,…,N. In this case, $\:{x}_{i\:}$ is an n-dimensional vector, with the corresponding $\:{y}_{i}\:$ being 1 for positive class samples and − 1 for negative class samples. The method to derive the decision functions for these two cases will be explained. Determining SVM for cases with linear and separability Support Vector Classification (SVC) distinguishes itself by introducing the concept of “margin,” representing the gap in between the separating hyperplane and the closest samples. It selects the hyperplane that maximizes this margin to ensure better predictive performance for future data as shown in Fig. 5. In the case of linear separability, every hyperplane f($\:{x}_{i}$) must satisfy the condition:

2 $$\:\mathrm{f}\left({x}_{i}\right)={{w}^{t}x}_{i}+b\ge\:1,{y}_{i}=1$$

3 $$\:\mathrm{f}\left({x}_{i}\right)={{w}^{t}x}_{i}+b\le\:-1,{y}_{i}=-1$$

Here, *w* represents the normalized weight vector with dimensions matching $\:{x}_{i}$, and *b* is the normalized bias of the hyperplane. These two constraints can be unified into a single expression:

4 $$\:({{w}^{t}x}_{i}+b){y}_{i}\ge\:1$$

It is important to note that normalizing *w* and *b* ensures that f(* x*) evaluates to either 1 or -1 when 𝑥 is on the boundary. Consequently, the gap between the two parallel hyperplanes can be expressed as:

5 $$\:\mathrm{M}\mathrm{a}\mathrm{r}\mathrm{g}\mathrm{i}\mathrm{n}\:=\:2\frac{\left|{w}^{t}\mathrm{x}+b\right|}{\|w\|}=2\:\frac{1}{\|w\|}$$

### Support vector machines for regression (SVR)

Unlike classical regression analysis, which aims to determine a function *f*(*x*) that minimizes the difference between observed and predicted values across all training data, SVM focuses on achieving optimal generalized performance by minimizing the generalization error limit, which balances the complexity of the model with the training error^69^. SVM has been expanded to solve regression tasks using the dataset $\:D={\left\{\left({x}_{i},{y}_{i}\right)\right\}}_{\mathrm{i}=1}^{N}$, generated from a latent function, where $\:{x}_{i}$ represents the input vector and $\:{y}_{i}$ corresponds to the output. *N* indicates the total samples’ number. In the regression process, the training set is represented as follows:

6 $$\:\mathrm{Z}\:=\:\left\{\right({x}_{1},{y}_{1}),\:({x}_{2},{y}_{2}),\dots\:,\:({x}_{m},{y}_{m}\left)\right\}\:$$

In this case, $\:{x}_{m}\:$ refers to the independent variable, while $\:{y}_{m}$ represents the dependent variable in the regression model.

7 $$\:{y}_{i}=\:\sum\:_{i=1}^{n}({\alpha\:}_{i}-{\beta\:}_{i})\:\times\:\left(x,\:{x}_{1}\:\right)+b\:$$

Here, *b* denotes the bias, while $\:{\alpha\:}_{i}$ and $\:{\beta\:}_{i}$ are the multipliers of Lagrange. For nonlinear cases, the function of kernel is introduced into the modified equation as follows:

8 $$\:{y}_{i}=\:\sum\:_{i=1}^{n}({\alpha\:}_{i}-{\beta\:}_{i})\:\times\:K\left({x}_{i},\:{x}_{j}\:\right)+b$$

Here, $\:K\left({x}_{i},\:{x}_{j}\:\right)\:$ represents the Kernel function.

### SVM kernel function

Kernel functions facilitate nonlinear transformations by mapping nonlinearly separable data into a higher-dimensional feature space, where the data becomes linearly separable^71^. Moreover, kernel functions need to meet the requirements outlined by Mercer’s theorem^72^. The most utilized kernel functions are shown in Tables 5 and 6^23^.

**Table 5:** Widely used SVM kernel functions.

Kernel name	Kernel function formula	Description
Linear kernel	K ($\:{x}_{i},{x}_{j})=({x}_{i}.{x}_{j})$	The Linear Kernel is the most basic Kernel function, defined by the inner product $\:({x}_{i},{x}_{j})$
Polynomial kernel	$\:\mathrm{K}\:({x}_{i},{x}_{j})=\:{({x}_{i}.{x}_{j}+1)}^{p}$	The Polynomial Kernel is a non-stationary kernel, ideal for problems with normalized training data. Commonly used degrees are 𝑑 = 2(quadratic) and 𝑑 = 3 (cubic), as higher degrees often lead to overfitting in machine learning tasks.
Gaussian kernel or radial basis function (RBF)	K ($\:{x}_{i},{x}_{j})={\mathrm{e}}^{\frac{-{\|{\mathrm{x}}_{\mathrm{i}}-{\mathrm{x}}_{\mathrm{j}}\|}^{2}}{2{{\upsigma\:}}^{2}}}$ or K ($\:{x}_{i},{x}_{j})={\mathrm{e}}^{-\gamma\:}{\left({x}_{i}-{x}_{j}\right)}^{2}$	In the Gaussian Kernel, the parameter $\:\gamma\:$ significantly influences the Kernel’s effectiveness. If y is set too high, the exponential function will approximate linear behavior, causing the higher-dimensional projection to lose its ability to represent non-linear relationships.

**Table 6:** SVM classifiers available in the MATLAB classification learner toolbox.

Classifier types	Classifier description from MATLAB classification learner toolbox
Linear SVM	Use a Linear Kernel to create a straightforward separation between classes. This is the simplest SVM to interpret.
Quadratic SVM	Utilizes the Quadratic Kernel.
Cubic SVM	Utilizes the Cubic Kernel.
Fine Gaussian SVM	Creates detailed class separations using a Gaussian Kernel, with the Kernel scale defined as sqrt(P)/4, where P is the count of predictors.
Medium Gaussian SVM	Creates less detailed separations compared to a Fine Gaussian SVM by using a Gaussian Kernel with the Kernel scale set to sqrt(P) where P is the number of predictors.
Coarse Gaussian SVM	Creates broad distinctions between classes by employing a Gaussian Kernel with the Kernel scale set to sqrt(P)*4, where P denotes the number of predictors.

### Classification learner app

MCLA offers a user-friendly platform for developing and accessing machine learning classifiers. It includes a wide selection of methods such as Decision Trees, Discriminant Analysis, SVM, Neural Networks, Ensemble approaches, Naïve Bayes, and KNN^23,73^. To avoid overfitting, the data was split into 70% for testing, 15% for training, and 15% for validation. Through comparative analysis based on prediction speed, accuracy and training time, the findings revealed that the SVM outperformed the other techniques, proving to be the most effective model for the classification objectives in this research. Prior to classifier training and evaluation, all extracted wavelet-based features are subjected to a normalization stage to ensure numerical stability and equitable feature contribution. Specifically, min–max normalization is applied on a feature-wise basis, scaling each feature to the range [0,1]. This step is particularly important given that the extracted features originate from different frequency bands and decomposition levels and may therefore exhibit varying numerical magnitudes. By preserving the relative distribution of the wavelet energy features while preventing dominance by higher-amplitude components, the normalization process enhances the robustness and convergence behavior of the SVM classifier. The normalized feature set is then used consistently for fault detection, classification, and location tasks under all operating conditions, including non-fault disturbances and scenarios with integrated distributed generation.

### Regression learner app

MRLA, available in MATLAB, provides an intuitive interface for automatically training and validating various regression models, including Gaussian process regression, Linear regression, support vector machines, decision trees, and tree ensembles, while also facilitating data import, true selection, model training, and result evaluation^74^. The models were evaluated and refined by analyzing residuals, R-squared values, Root Mean Square Error (RMSE) and Mean Square Error (MSE) to identify the most accurate predictions^75^.

## Modeling of renewable energy sources

**Fig. 6:**
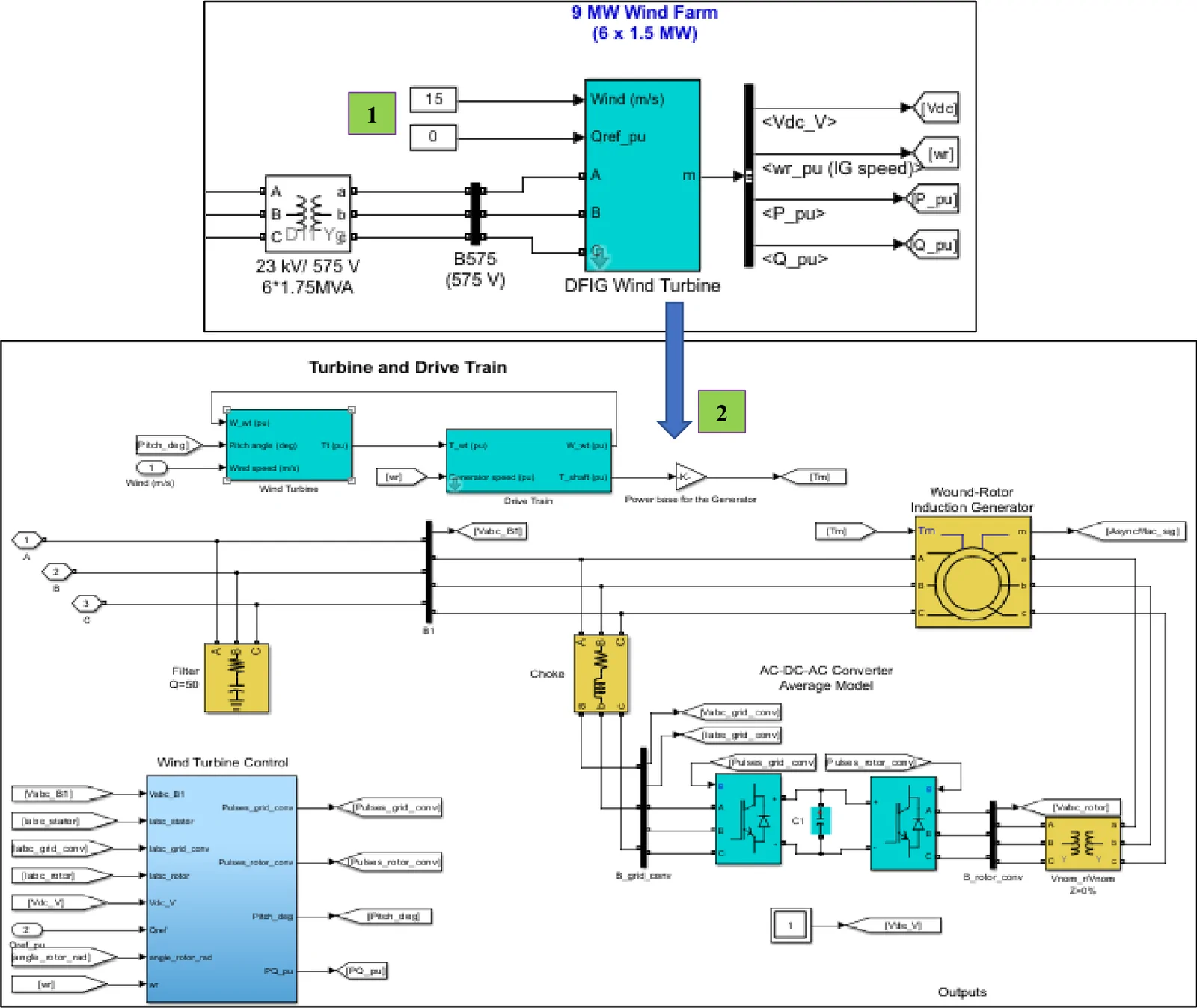
Detailed simulink-based wind generation architecture.

To evaluate the robustness of the proposed IoT–DWT–SVM protection framework under renewable-integrated operating conditions, photovoltaic (PV) and wind generation units were incorporated into the IEEE 16-bus distribution system using detailed MATLAB/Simulink models. The objective was to assess whether the proposed protection methodology maintains reliable fault detection, classification, and location performance under the modified network operating conditions introduced by distributed generation (DG). The wind generation system was modeled as a doubly-fed induction generator (DFIG)-based wind farm with a total installed capacity of 9 MW, consisting of six aggregated wind turbine units rated at 1.5 MW each. The DFIG-based wind farm was interfaced with the distribution network through an appropriate step-up transformer, allowing the exchange of both active and reactive power with the grid while representing the electromechanical behavior incorporated within the adopted MATLAB/Simulink model. The detailed implementation of the adopted DFIG-based wind generation model is shown in Fig. 6.

**Fig. 7:**
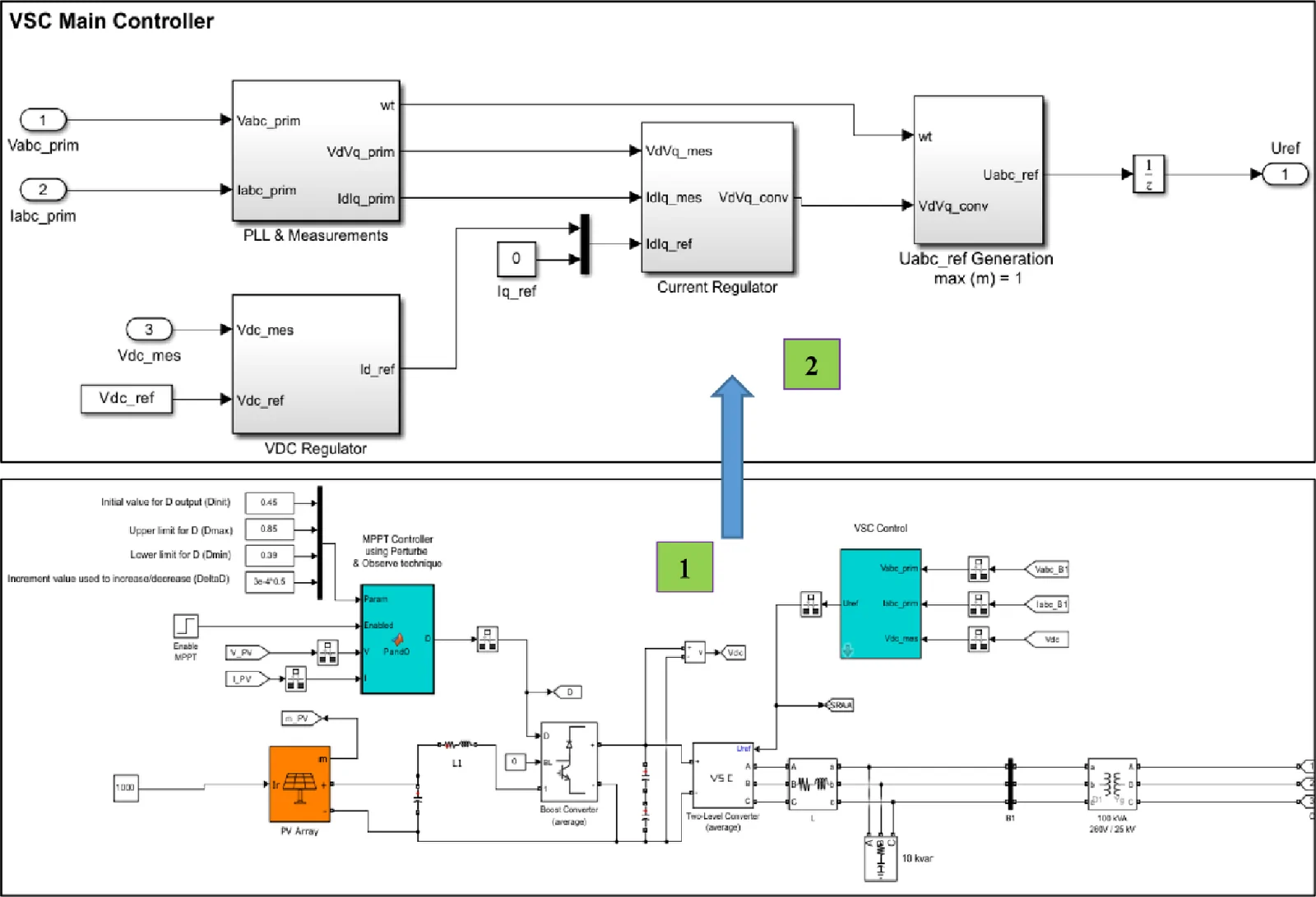
Detailed simulink-based PV generation architecture.

Similarly, the photovoltaic generation system was implemented using the corresponding MATLAB/Simulink PV generation architecture with a total installed capacity of 9 MW. The PV source was connected to the distribution network through a power electronic interface and transformer arrangement consistent with the adopted simulation model. The integration of the PV system enabled the investigation of protection performance under inverter-interfaced renewable generation scenarios characterized by modified fault-current characteristics and varying operating conditions. The DG models employed in this study were intended to provide representative renewable-integrated operating conditions for evaluating the proposed protection methodology rather than to conduct a dedicated analysis of converter protection design. The detailed MATLAB/Simulink implementation of the adopted photovoltaic generation model is presented in Fig. 7. Therefore, although the adopted models capture the dynamic interaction between renewable sources and the distribution network within the simulation environment, advanced investigations related to detailed inverter protection behavior, manufacturer-specific control implementations, low-voltage ride-through functionalities, explicit converter current-limiting strategies, and DG protection coordination were beyond the scope of the present work. Accordingly, the primary focus of this study was to assess whether the proposed DWT-based feature extraction and SVM-based decision framework remained effective in detecting, classifying, and locating faults in the presence of distributed renewable generation.

The simulation results demonstrated that the proposed intelligent protection scheme maintained high fault detection, classification, and location performance under both conventional operating conditions and the investigated renewable-integrated scenarios, indicating its potential applicability to active distribution networks while remaining within the assumptions of the adopted simulation models. The photovoltaic and wind generation units incorporated in this study were modeled to represent the influence of distributed generation on the operating conditions of the IEEE 16-bus distribution network and to evaluate the robustness of the proposed DWT–SVM-based protection framework under renewable-integrated scenarios. Accordingly, the adopted DG models were intended to reproduce the electrical interaction between distributed generation and the network within the investigated simulation environment rather than to provide a detailed implementation of inverter control algorithms or protection functions. Consequently, inverter current-limiting behavior, fault ride-through capability, and DG protection coordination were represented by the default control and protection characteristics embedded in the adopted MATLAB/Simulink models and were not treated as independent variables in the present study. Therefore, the conclusions of this work should be interpreted within the scope of the adopted simulation models. A comprehensive investigation of detailed inverter control strategies, manufacturer-specific protection functions, converter current-limiting behavior, and coordinated DG protection would require dedicated electromagnetic transient studies and is therefore identified as an important direction for future research.

## Case study system

**Fig. 8:**
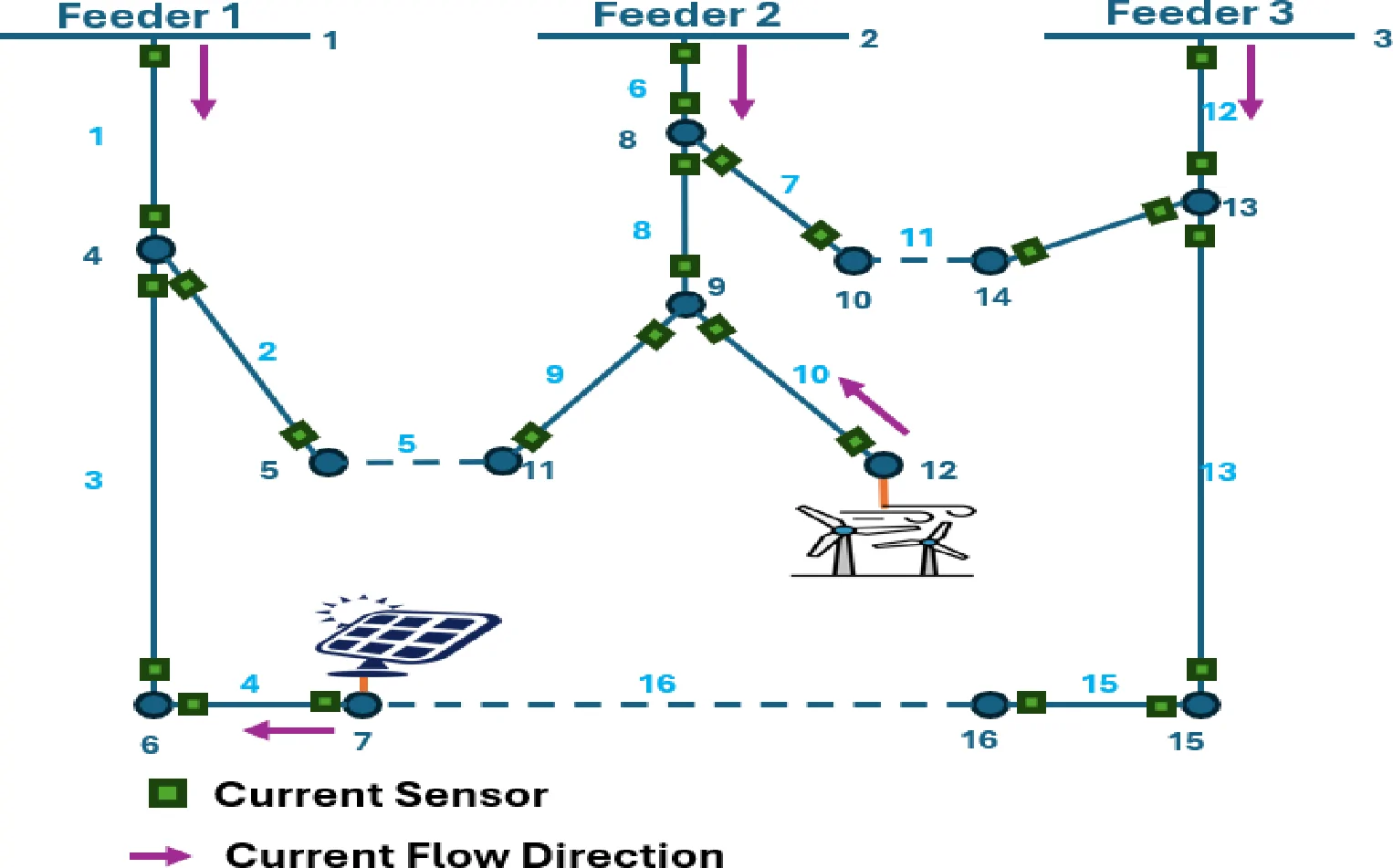
IEEE-16 bus system with DGs.

An IEEE 16-bus distribution system featuring 3 feeders, each with a base power and voltage of one hundred MVA and 23 kV respectively^41^. It is evident that the network configuration for Feeder 1 and Feeder 3 is identical, thus assuming that the simulation results for both feeders are equivalent. The system’s total load is 28.7 MW and 17.3 MVAr, with accumulative reactive power injection from various loads reaching 11.4 MVAr. Details regarding the line and bus data of this distribution system are provided in^41^. To complete the study of different scenarios on the IEEE-16 bus system, renewable distributed energy sources, including solar at Bus 7 and wind at Bus 12, are incorporated, as depicted in Fig. 8. IEEE-16 bus system is modeled by using MATLAB/Simulink tool. Various scenarios are simulated, including:

- Overload: 1.25% and 1.5%.
- Load switching and capacitor bank operation.
- Types of fault: Grounded Line Fault (L-G), Double Line Fault (LL), Double Line to Ground (LL-G), Triple Line Fault (3 L), and Triple Line to Ground Fault (3 L-G).
- Validation of high distributed generation penetration (9 MW photovoltaic and 9 MW wind generation).
- Resistance of fault: 0.1 Ω: 50 Ω.
- Multiple fault current inception angles: 0°, 30°, 45°, 60°, 90°, 120°, and 150°.
- Multiple fault locations: 10%: 90% from busbar.

## Results and comparative analysis for detection, classification and location scheme

### Fault detection

Table 7 presents representative results from the comprehensive validation tests conducted under different fault conditions, including variations in fault location, fault type, and fault inception angle. In all investigated cases, the proposed DWT–SVM framework correctly detected and classified the faults. Furthermore, various non-fault switching events were simulated, and the proposed method successfully identified all of them as normal operating conditions. These results demonstrate the robustness of the proposed framework and its ability to reliably distinguish faults from non-fault disturbances while maintaining consistent performance under diverse operating scenarios.

**Table 7:** Fault classification samples based various fault types and normal switching cases to train algorithm.

Simulated case	Fault resistance ($\:\boldsymbol{\Omega\:}$)	Fault inception angle (φ°)	Fault location	Maximum absolute value for current detailed coefficients and Sum absolute value for current detailed coefficients				Fault classification
				$\:\left|\boldsymbol{S}\boldsymbol{a}\right|$×10^3^ $\:\left|\boldsymbol{M}\boldsymbol{a}\right|$×10^3^	$\:\left|\boldsymbol{S}\boldsymbol{b}\right|\times\:10$ ^3^	$\:\left|\boldsymbol{S}\boldsymbol{c}\right|\times\:10$ ^3^	$\:\left|\boldsymbol{S}\boldsymbol{g}\right|\times\:10$ ^3^	
L-GF	10	30°	**50%**	**277.049**	19.662	20.015	**257.613**	L-GF(AG)
				**2.752**	0.195	0.198	**2.559**	
L-GF	15	30°	**15%**	19.601	**198.783**	20.015	**179.285**	L-GF(BG)
				0.195	**1.979**	0.198	1.786	
L-GF	10	60°	**25%**	19.601	19.663	**277.292**	**257.759**	L-GF(CG)
				0.195	0.195	**2.760**	**2.565**	
L-GF + DG	10	60°	**25%**	**221.408**	56.161	53.208	**188.811**	L-GF(AG)
				**2.205**	0.556	0.542	**1.879**	
L-GF + DG	15	90°	**35%**	57.171	**146.399**	53.206	126.913	L-GF(BG)
				0.564	**1.453**	524	1.259	
L-GF + DG	25	90°	**45%**	57.171	56.158	**84.307**	**78.319**	L-GF(CG)
				0.564	0.556	**0.84**	**0.78**	
LL-F	35	45°	**25%**	**84.632**	**87.668**	20.015	9.638	LL-F(AB)
				**0.840**	**0.873**	0.198	0.114	
LL-F	10	120°	**75%**	19.600	**238.419**	**239.943**	9.637	LL-F(BC)
				0.195	**2.374**	**2.390**	0.114	
LL-F + DG	15	150°	**35%**	**158.030**	**126.576**	53.206	16.377	LL-F(AB)
				**1.553**	**1.26**	0.524	0.189	
LL-F + DG	25	30°	**90%**	**65.676**	56.151	**97.125**	16.373	LL-F(AC)
				**0.655**	0.556	**0.965**	0.188	
LL-GF	45	45°	**20%**	**81.716**	**81.670**	20.015	**61.958**	LL-GF(ABG)
				**0.813**	**0.814**	0.198	**0.614**	
LL-GF	25	150°	**55%**	**129.448**	19.662	**127.729**	**109.813**	LL-GF(ACG)
				**1.283**	195	**1.268**	**1.094**	
LL-GF + DG	35	90°	**10%**	**61.439**	56.163	**72.675**	**61.011**	LL-GF(ABG)
				**0.61**	0.556	**0.722**	**0.605**	
LL-GF + DG	15	**0**°	**75%**	57.170	**165.773**	**144.560**	**126.416**	LL-GF(BCG)
				0.564	**1.649**	**1.438**	**1.259**	
3 L-F	50	45	**13%**	**75.621**	**75.580**	**74.741**	9.638	3 L-F (ABC)
				**0.754**	**0.753**	**0.74**	0.114	
3 L-F + DG	5	120°	**78%**	**452.717**	**450.577**	**451.247**	16.374	3 L-F (ABC)
				**4.471**	**4.574**	**4.497**	0.188	
3 L-GF	0.1	60°	**67%**	**276.005**	**276.5**	**274.924**	**51.126**	3 L-GF(ABCG)
				**2.742**	**2.75**	**2.737**	**0.511**	
3 L-GF + DG	2	90°	**46%**	**860.816**	**862.802**	**852.744**	**96.457**	3 L-GF(ABCG)
				**8.574**	**8.559**	**8.509**	**0.926**	
Cap. Sw.	–	–	–	24.925	25.038	26.594	9.637	Normal case
				0.248	0.25	0.261	0.114	
Load Sw.	–	–	–	47.447	47.917	47.803	9.637	Normal case
				0.474	0.476	0.476	0.114	
Overload	–	–	–	24.731	24.757	24.880	11.443	Normal case
				0.246	0.246	0.247	0.135	
Base case (no-fault)	–	–	–	19.6	19.662	20.014	9.637	No-fault (normal mode)
				0.195	0.195	0.198	0.114	

Cap. Sw. = Capacitor Switching at 40%, and 100% of rated 3.7 MVArs; Load Sw.= Load Switching at 20%, and 40% of max. load 5.02 Mw.

**Fig. 9:**
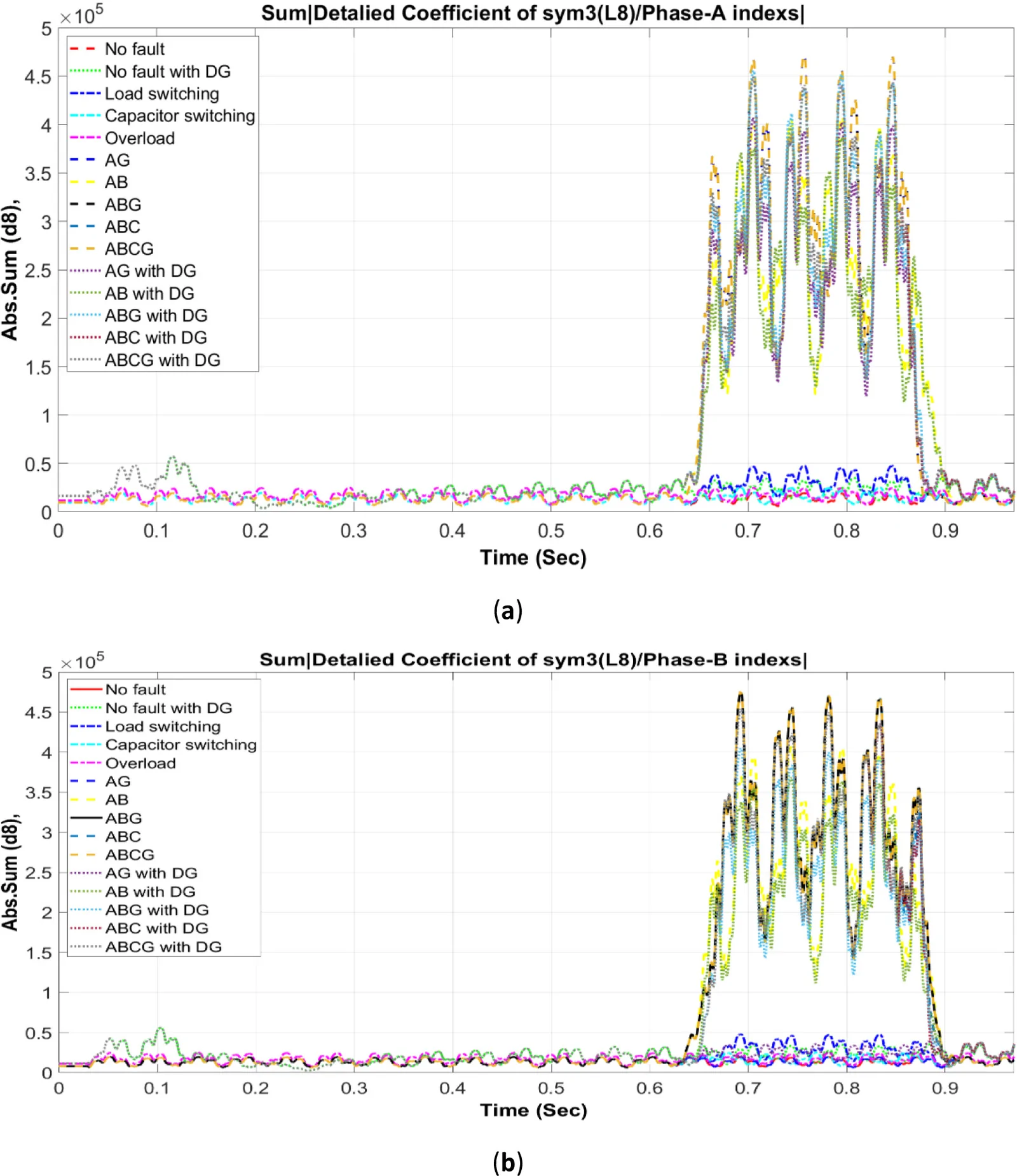

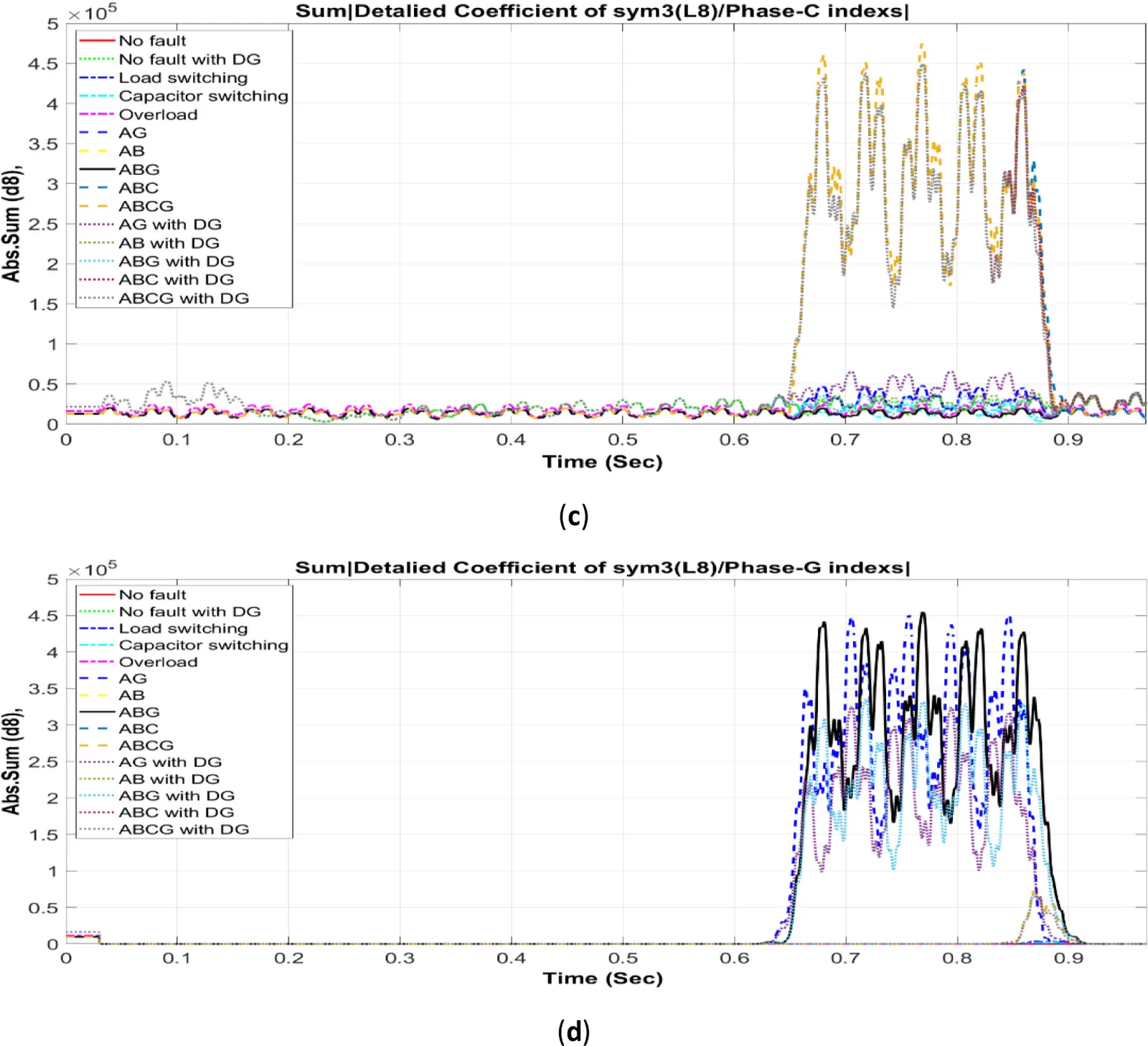
Calculated total of all absolute for current detailed coefficient at sym3 with Level 8 (Sa), and Geometric depiction of the suitable classifier for different case studies with and without DGs. (**a**) Total of all absolute for phase-A current detailed coefficient at sym3 (Level 8). (**b**) Total of all absolute for phase-B current detailed coefficient at sym3 (Level 8). (**c**) Total of all absolute for phase-C current detailed coefficient at sym3 (Level 8). (**d**) Total of all absolute for Ground current detailed coefficient at sym3 (Level 8).

Figure 9 illustrates the sum of the absolute values of the detail coefficients (Sa) of the Sym3 wavelet at level 8 for Phase-C current signals. Fault conditions produce pronounced high-frequency energy components compared to non-fault disturbances, including load switching, capacitor switching, and overload events, both with and without distributed generation. This clear separation confirms the discriminative capability of the extracted features and explains the high classification performance of the proposed SVM-based classifier.

### ThingSpeak-based monitoring of current phases

**Fig. 10:**
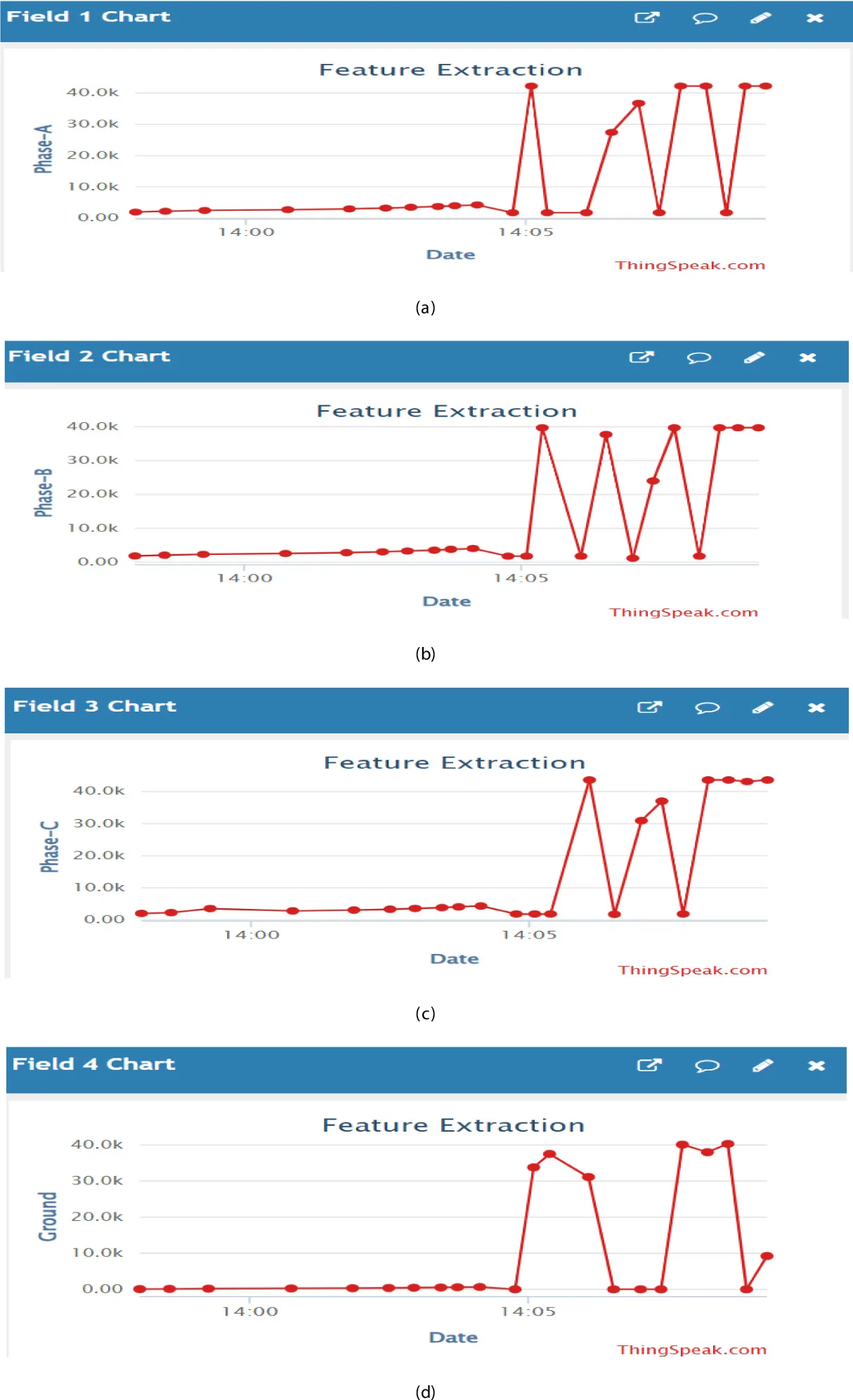
Real-time monitoring of current phases on the ThingSpeak server. (**a**) The maximum feature from (D8) of phase-A is transmitted to the cloud. (**b**) The maximum feature from (D8) of phase-B is transmitted to the cloud. (**c**) The maximum feature from (D8) of phase-C is transmitted to the cloud. (**d**) The maximum feature from (D8) of Ground is transmitted to the cloud.

Figure 10 illustrates the cloud-assisted monitoring framework adopted in this study. Following the DWT analysis, the maximum value of the detail coefficient extracted using the Sym3 mother wavelet at the eighth decomposition level (D8) was selected as a representative fault-sensitive feature due to its superior discrimination capability identified during the feature evaluation process. This feature was transmitted to the ThingSpeak cloud platform through its application programming interface (API), enabling remote visualization and monitoring of abnormal operating conditions in near real time. The uploaded feature data were subsequently accessed by the SVM-based decision module for fault detection, classification, and location purposes within the proposed protection framework. The pronounced spikes observed in the ThingSpeak records correspond to the occurrence of abnormal events and demonstrate the capability of the adopted architecture to communicate fault-related information beyond the local protection environment.

It should be emphasized that the objective of the implemented IoT layer was to demonstrate the feasibility of integrating cloud-based monitoring with the proposed DWT–SVM methodology and to enhance situational awareness in smart distribution networks. Therefore, ThingSpeak was utilized as a proof-of-concept platform for remote data accessibility rather than as a comprehensive field-deployable communication infrastructure.

### Practical considerations of the IoT layer

The IoT implementation presented in this work was intended to demonstrate the integration of cloud-enabled monitoring within the proposed protection framework. Consequently, several practical aspects associated with large-scale deployment of IoT-based protection systems were not explicitly investigated. These include communication latency, bandwidth limitations, time synchronization, cybersecurity requirements, communication reliability,, and scalability under extensive sensor deployment. Although these factors may influence the performance of practical implementations, they do not alter the primary objective of the present study, which was to evaluate the effectiveness of the DWT–SVM methodology and demonstrate the feasibility of incorporating remote monitoring capabilities into distribution system protection. Future investigations will address these communication-related challenges through the adoption of advanced IoT architectures, edge-computing strategies, and experimentally validated communication platforms.

### Timing analysis of the local protection decision process

Protection systems require prompt fault identification and decision-making to ensure selective isolation and maintain system stability. In the proposed methodology, the three-phase current signals were sampled at 20 kHz. Consequently, at the nominal system frequency of 50 Hz, one fundamental cycle corresponds to 20 ms and contains 400 samples. Following fault inception, a one-cycle post-event window was utilized for DWT-based feature extraction. This observation window constitutes the dominant component of the local protection decision time. The protection decisions reported in this work were obtained using the extracted DWT features and pre-trained SVM models. Although explicit execution-time measurements were not performed in the present study, published studies have reported that wavelet-based feature extraction and SVM inference are typically completed within the millisecond range on modern computing platforms. In practical implementations, the overall operating time of similar intelligent protection schemes is typically dominated by the data acquisition window, while the machine-learning decision process introduces only a relatively small computational burden. Table 8 summarizes representative timing components commonly encountered in intelligent protection frameworks. These values are provided solely to illustrate the expected order of magnitude of the individual stages and should not be interpreted as experimentally validated operating times of the proposed implementation. Since the cloud platform is not included in the protection decision path, only the timing components associated with the local protection algorithm are considered in the following analysis.

**Table 8:** Representative timing components of the proposed local protection framework.

Local protection stage	Representative time
Data acquisition (400 samples)	20 ms (one cycle at 50 Hz)
DWT decomposition (400 samples, Sym3, L8)	2.6 ms
Feature extraction (maximum D8)	0.2 ms
SVM classification	0.3 ms
SVM fault location	0.2 ms
Local decision logic	1.0 ms
Total local protection time	≈ 24.3 ms
Cloud communication latency (ThingSpeak)	Excluded from the local protection decision path

*Note: Sampling period = 50 µs (20 kHz sampling frequency). The reported timing values correspond to implementation-based estimates derived from the adopted MATLAB computational workflow.

The timing values reported in Table 8 represent implementation-based estimates derived from the computational characteristics of the adopted MATLAB implementation and the corresponding signal-processing workflow. They are provided to illustrate the relative computational contribution of each processing stage and should not be interpreted as experimentally measured execution times on a dedicated hardware platform.

Accordingly, the expected local protection decision time of the proposed DWT–SVM framework is dominated by the one-cycle observation window and is estimated to be approximately 24.3 ms under the adopted local processing architecture. It should be emphasized that the ThingSpeak platform was employed exclusively to demonstrate cloud-assisted monitoring, visualization, and remote accessibility of fault-related information. The protection decisions presented in this work were generated entirely within the local DWT–SVM framework and were independent of Internet-based communication for relay tripping purposes. Consequently, communication latency associated with the cloud platform does not influence the execution of the local protection algorithm or the reported protection performance. Experimental measurement of the complete end-to-end operating time using embedded hardware platforms (e.g., FPGA- or DSP-based controllers) under realistic communication environments constitutes an important direction for future research and is required for comprehensive validation of the proposed protection framework.

### Fault classification

**Fig. 11:**
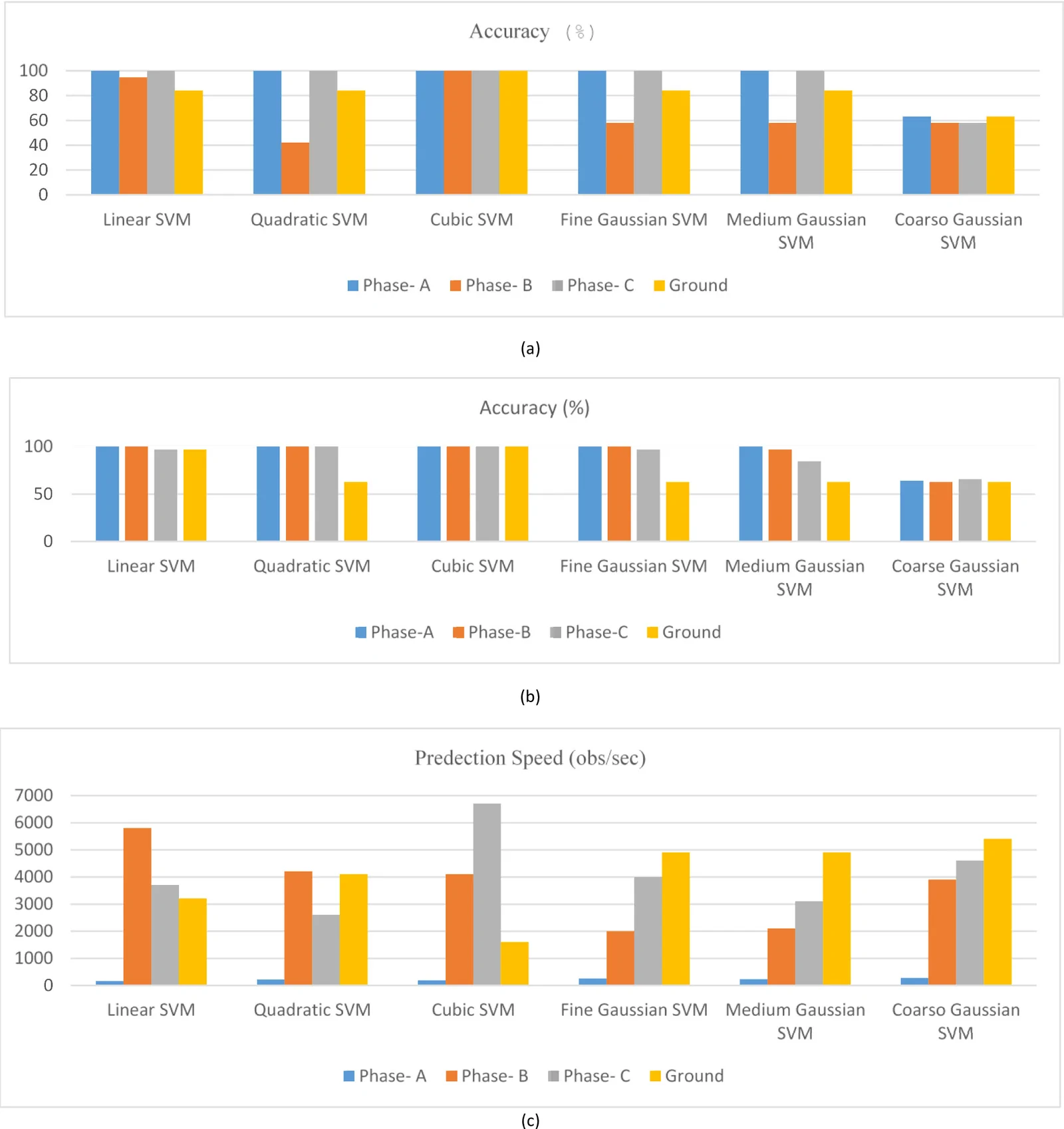

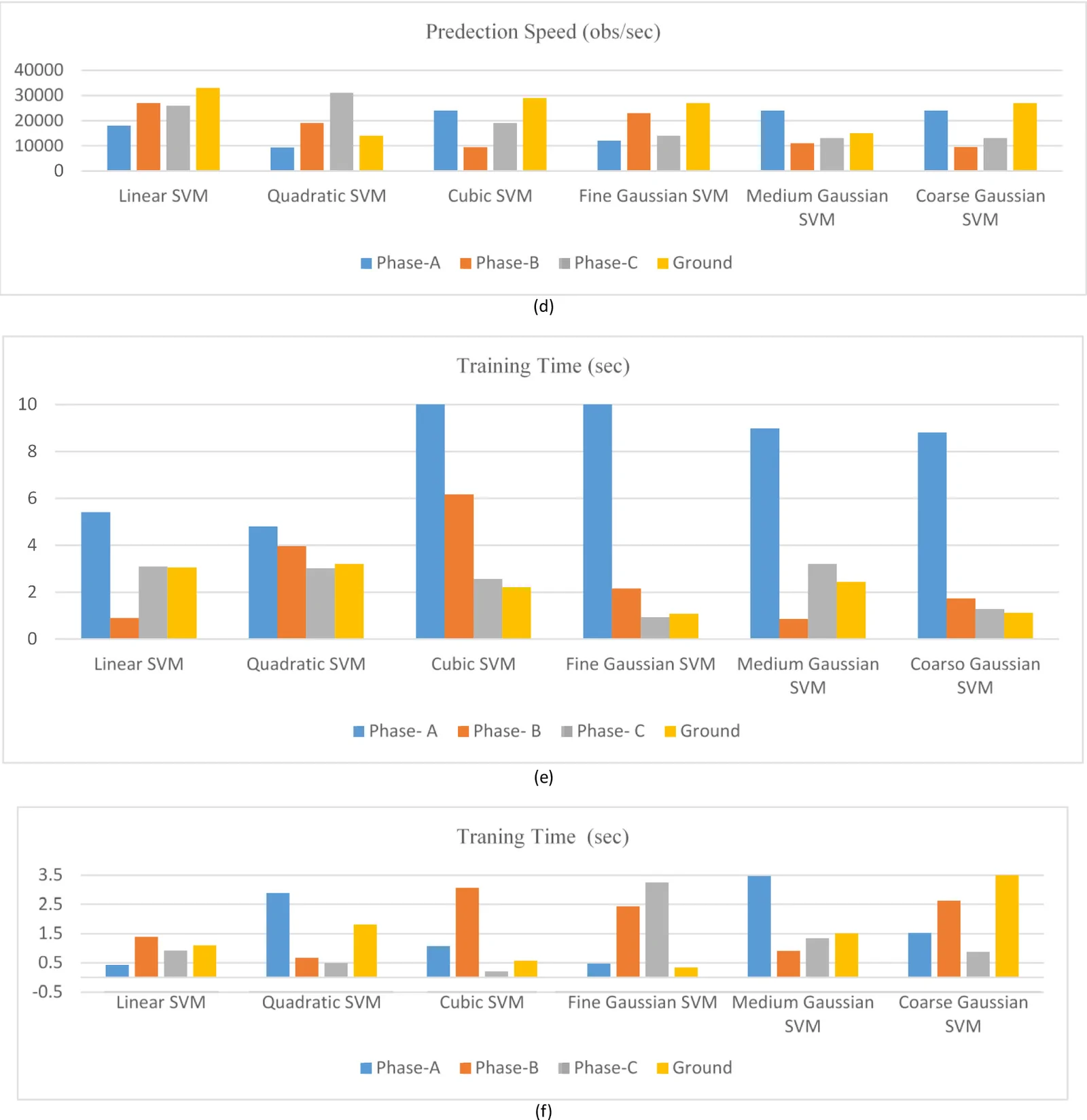
Comparison of fault classification accuracy, prediction speed, and training time for six SVM models across all phases in IEEE 16 bus with and without DGs. (**a**) Fault classification accuracy per phase in distribution system without DGs. (**b**) Fault classification accuracy per phase in distribution system with DGs. (**c**) Fault classification prediction speed per phase in distribution system without DGs. (**d**) Fault classification prediction speed per phase in distribution system with DGs. (**e**) Fault classification training time per phase in distribution system without DGs. (**f**) Fault classification training time per phase in distribution system with DGs.

**Fig. 12:**
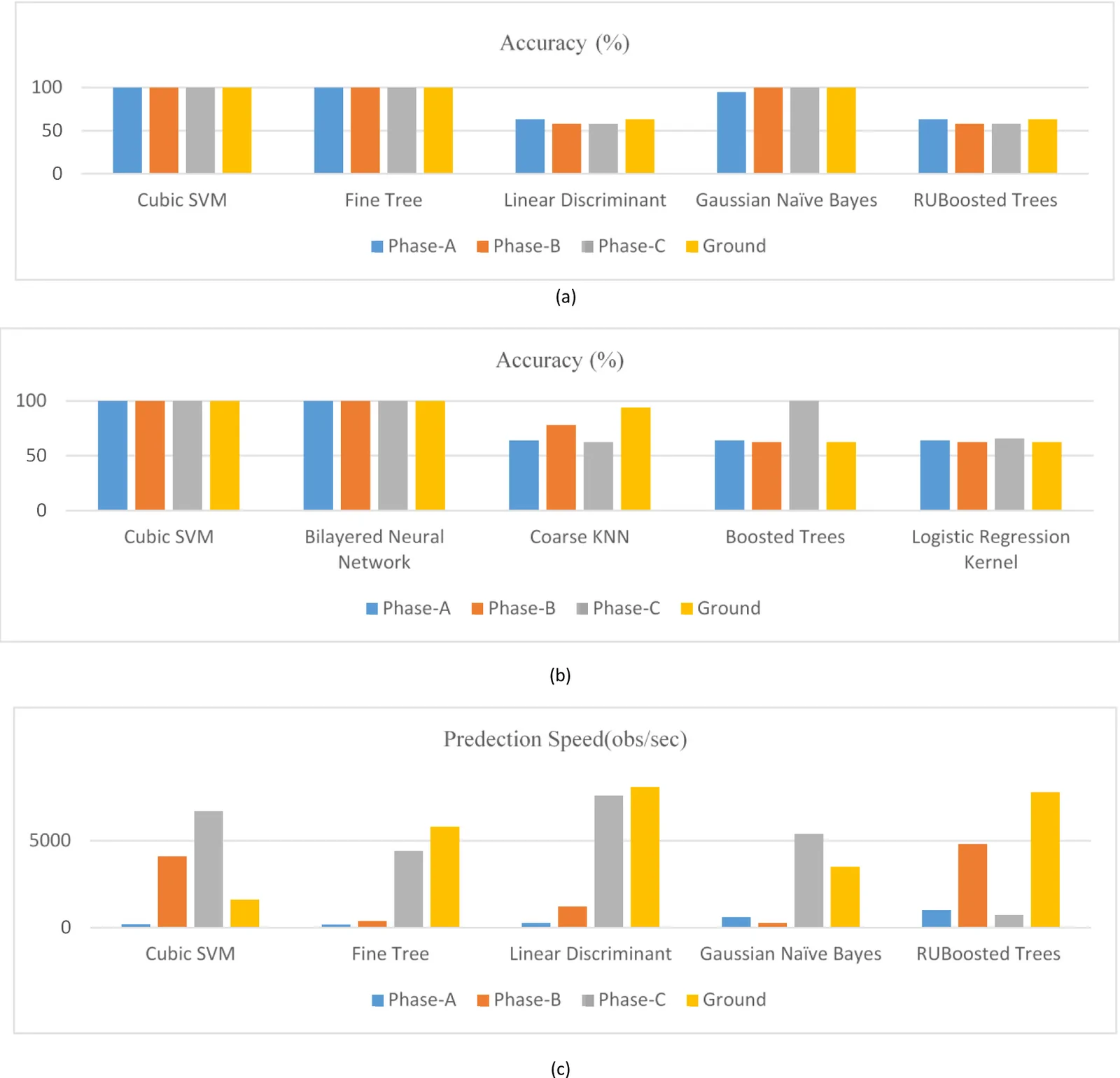

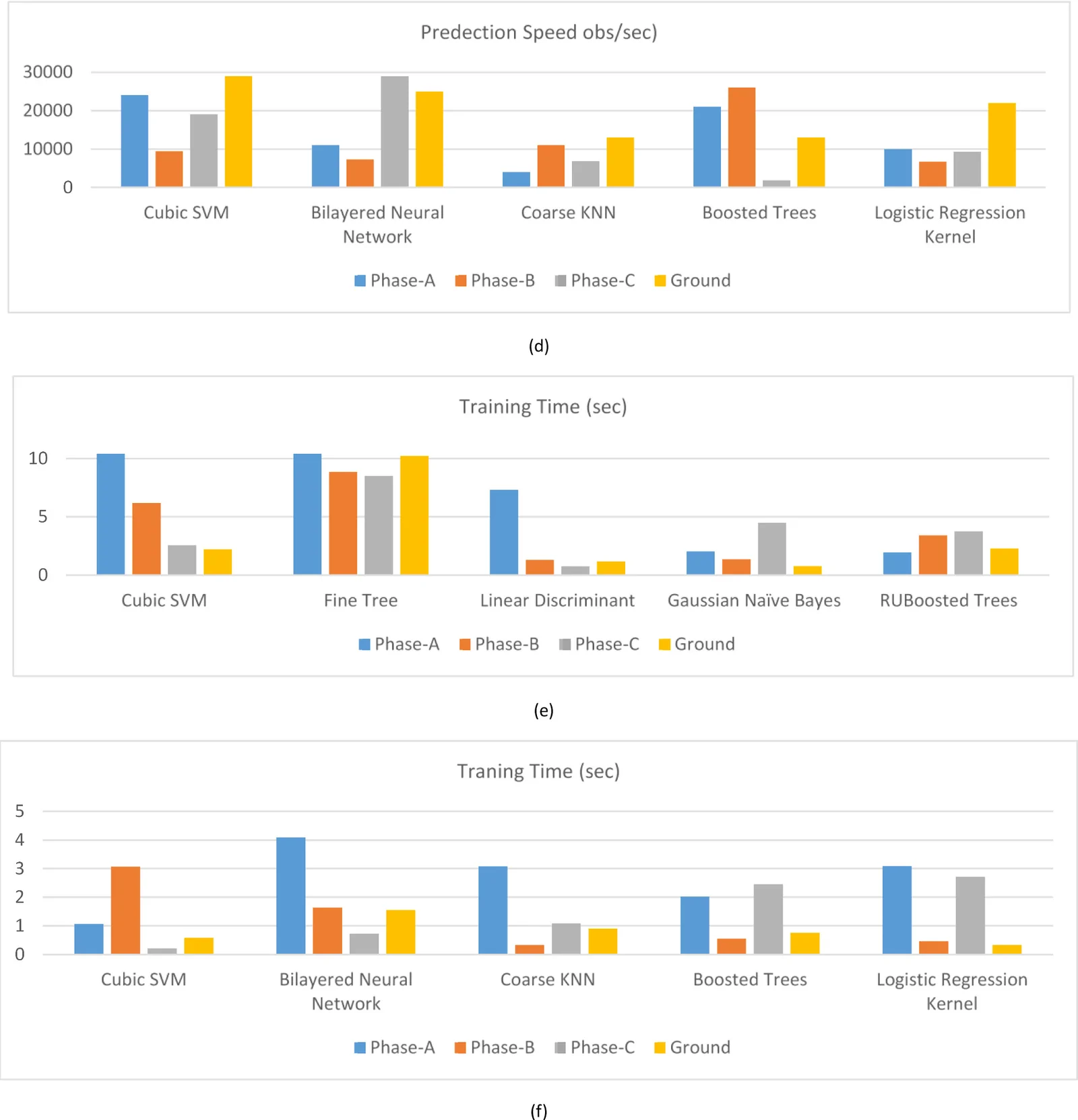
Comparison of fault classification accuracy, prediction speed, and training time for 6 ML algorithms across all phases in IEEE 16 bus with and without DGs. (**a**) Fault classification accuracy per phase in distribution system without DGs. (**b**) Fault classification accuracy per phase in distribution system with DGs. (**c**) Fault classification prediction speed per phase in distribution system without DGs. (**d**) Fault classification prediction speed per phase in distribution system with DGs. (**e**) Fault classification training time per phase in distribution system without DGs. (**f**) Fault classification training time per phase in distribution system with DGs.

Figures 11 and 12 show the performance of various classification algorithms evaluate for fault classification in distribution systems both with and without DGs. In both scenarios, Cubic SVM consistently outperformed all other classifiers, achieving 100% accuracy across all phases, along with the highest prediction speeds and low training times, especially in the presence of DGs where its computational efficiency significantly improved.

The superior classification performance achieved by the Cubic SVM can be attributed to its ability to model nonlinear relationships between the extracted DWT features and the underlying fault categories. Fault-generated transients often exhibit complex patterns that cannot be completely separated using linear decision boundaries. The higher-order kernel employed by the Cubic SVM provides greater flexibility in capturing these nonlinear characteristics, resulting in improved classification accuracy while maintaining acceptable computational complexity.

Although several machine-learning algorithms achieved competitive classification accuracies, noticeable differences were observed in prediction speed and training time. These differences highlight the importance of considering both classification performance and computational efficiency when selecting a protection algorithm. In practical protection applications, rapid decision-making is essential; therefore, models that simultaneously provide high accuracy and low computational burden are generally preferred.

### Fault location

The performance of fault location was thoroughly evaluated using various SVR models within MATLAB’s Regression Learner App. As shown in Tables 9, 10 and 11, the Linear SVM model consistently outperformed other regression techniques by achieving the lowest Root Mean Square Error (RMSE) across all major fault categories, including L-GF, LL-F, LL-GF, 3 L-F, and 3 L-GF. This high level of accuracy was maintained in both scenarios with and without the integration of DGs underscoring the robustness and generalization capability of the Linear SVM model. For fault location, the model achieved exceptional precision, with accuracy ranging from 97.95% to 99.88% without DGs, and from 97.87% to 98.45% with DGs, confirming its reliability and effectiveness under varying network conditions.

The superior performance of the Linear SVM model suggests that the relationship between the selected DWT-based features and fault location exhibits a high degree of linear separability within the investigated operating scenarios. Unlike higher-order kernels, which may introduce unnecessary model complexity, the Linear SVM provided improved generalization capability and reduced prediction error. This observation highlights the effectiveness of the extracted wavelet features in preserving fault-location information while maintaining a computationally efficient regression framework suitable for practical protection applications.

**Table 9:** RMSE (%) for fault location without DGs.

Fault type	Algorithm					
	Linear SVM	Quadratic SVM	Cubic SVM	Fine Gaussian SVM	Medium Gaussian SVM	Coarse Gaussian SVM
A_G	1.7737	2.4428	1.8837	7.7705	3.4303	9.0487
B_G	1.0219	2.5122	2.319	1.8217	1.1736	5.6321
C_G	1.6180	2.4945	1.9806	4.9234	1.6243	7.0599
AB	3.6952	5.7895	5.4388	10.076	5.7794	5.9549
AC	0.84503	1.4753	2.7543	4.9803	1.8666	5.14
BC	1.16520	1.1764	2.97500	1.1764	2.9750	5.1708
ABG	0.11711	1.3799	1.48280	2.1971	1.5518	6.8985
ACG	0.793446	2.3507	1.92554	3.2413	1.9340	4.4111
BCG	0.138290	3.4696	8.59610	3.1600	2.6813	1.0207
ABC	2.053400	2.2231	2.24900	15.999	4.9832	6.547
ABCG	1.679500	2.1602	2.96900	5.8477	2.3278	3.053

**Table 10:** RMSE (%) for fault location with 9 MW wind farm @ B12.

Fault type	Algorithm					
	Linear SVM	Quadratic SVM	Cubic SVM	Fine Gaussian SVM	Medium Gaussian SVM	Coarse Gaussian SVM
A_G	1.7940	2.0973	3.5966	19.582	5.4794	4.6952
B_G	2.1885	5.1088	4.9557	13.196	5.0383	4.5458
C_G	2.2417	2.2947	2.4727	4.7889	2.4721	3.1761
AB	5.1683	6.6660	5.8861	6.9106	5.6426	11.303
AC	2.0932	2.9985	3.7779	8.6800	2.5169	2.1902
BC	1.5523	1.7408	2.4269	2.7487	9.4288	18.824
ABG	1.6017	2.3525	2.4496	5.5494	2.6772	2.8923
ACG	1.7656	3.8161	5.7693	24.041	10.914	4.5509
BCG	1.9535	2.1099	5.5793	9.9887	5.1317	4.4856
ABC	2.1294	2.5010	2.4920	5.7829	3.3300	2.6728
ABCG	1.7846	2.2541	2.4400	3.0869	1.8220	2.9570

**Table 11:** RMSE (%) for fault location with 9 MW PV array @ B7.

Fault type	Algorithm					
	Linear SVM	Quadratic SVM	Cubic SVM	Fine Gaussian SVM	Medium Gaussian SVM	Coarse Gaussian SVM
A_G	2.2076	6.4686	2.5677	4.4265	5.0530	3.4790
B_G	1.9518	2.8966	3.3776	6.8360	4.6446	5.2033
C_G	2.3566	7.2770	11.1580	12.670	7.0867	3.9037
AB	1.4349	1.9732	3.1656	3.2490	2.8363	1.4506
AC	1.8414	2.3226	2.7513	3.4728	3.1350	12.808
BC	2.6544	4.2231	6.5305	6.4957	4.4366	5.5501
ABG	1.5472	2.2163	2.4888	2.7769	2.1884	5.3574
ACG	2.2531	3.1893	3.0859	8.3499	5.4013	13.961
BCG	1.3540	1.8822	18.065	2.7726	2.3323	2.5148
ABC	2.6745	6.0753	19.802	5.3253	5.1762	4.1213
ABCG	1.9394	2.4612	5.5752	6.4524	4.1831	7.0173

The consistent superiority of the Linear SVM model across different fault categories suggests that the selected wavelet features preserve a stable relationship with fault location. This finding is particularly important because it indicates that highly accurate fault-location estimates can be achieved without resorting to excessively complex regression structures, thereby reducing computational requirements and improving suitability for real-time protection-support applications. The obtained results demonstrate that the effectiveness of the proposed protection framework is strongly influenced by the quality of the extracted wavelet features. The superior performance achieved using the Sym3 mother wavelet at the eighth decomposition level can be attributed to its enhanced capability to capture transient fault characteristics while preserving discriminative information relevant to different fault conditions. This indicates that appropriate feature selection plays a crucial role in improving both classification accuracy and computational efficiency. These results are particularly relevant for modern distribution systems experiencing increasing penetration of distributed energy resources, where conventional protection methods may encounter challenges due to bidirectional power flows and altered fault-current characteristics. The ability of the proposed framework to maintain high performance under both conventional and renewable-integrated operating conditions demonstrates its potential as an adaptive protection-support tool for future smart-grid environments.

In addition, the consistent performance of the selected SVM models under various operating conditions, including overload events, switching operations, and renewable-integrated scenarios, highlights the robustness and adaptability of the proposed methodology. These findings suggest that combining DWT-based feature extraction with SVM-based decision-making provides an effective framework for addressing the challenges associated with modern active distribution networks, where traditional protection techniques may experience degraded performance due to increasingly dynamic operating conditions. Overall, the obtained results demonstrate that the effectiveness of the proposed protection framework is not solely dependent on the machine-learning model itself but also on the quality of the extracted DWT features. The combination of appropriate wavelet decomposition and SVM-based learning enabled accurate discrimination between fault and non-fault events while maintaining high performance under renewable-integrated operating conditions. These findings confirm the suitability of the proposed framework for intelligent monitoring and protection applications in evolving distribution networks.

## Practical considerations and limitations

The present study was validated using datasets generated from detailed MATLAB/Simulink simulations of the IEEE 16-bus distribution system. Although a wide range of operating scenarios was considered, including different fault conditions, non-fault disturbances, and renewable energy integration, the present validation did not explicitly account for measurement noise, sensor inaccuracies, communication delays, or other practical uncertainties encountered in real distribution networks. Therefore, the reported performance metrics should be interpreted within the scope of the investigated simulation environment. Future work will focus on evaluating the proposed protection scheme using noisy measurements and experimentally acquired datasets to further assess its statistical robustness and practical applicability under real operating conditions.

From a practical perspective, measurement noise, sensor inaccuracies, communication disturbances, and data corruption may affect the quality of the extracted fault features and consequently influence the performance of machine-learning-based protection algorithms. The present study intentionally isolated these practical uncertainty sources in order to establish the baseline performance of the proposed DWT–SVM framework under controlled simulation conditions. This approach enables the intrinsic performance of the proposed methodology to be evaluated before introducing external uncertainty sources that may influence the observed performance. Accordingly, the reported classification and fault-location accuracies should be interpreted as baseline performance obtained under the investigated simulation environment rather than as direct indicators of field performance. Communication latency was not explicitly modeled because the cloud platform was designed exclusively for monitoring purposes, whereas all protection decisions were executed locally within the proposed DWT–SVM framework. Comprehensive robustness assessment under stochastic measurement errors, communication impairments, hardware-related uncertainties, and practical implementation constraints represents the next stage of validation and will be addressed using experimentally acquired datasets and realistic communication environments.

In addition, the study was conducted using a single benchmark distribution network topology. Although the IEEE 16-bus system provides a representative platform for evaluating protection algorithms, further validation on larger and more complex distribution networks with varying configurations and operating characteristics would provide additional evidence regarding the scalability and generalization capability of the proposed framework. Finally, Although the computational workflow of the proposed algorithm was evaluated within the adopted simulation environment, experimental measurement of end-to-end execution time using an embedded hardware platform (e.g., FPGA- or DSP-based controller) or a real-time digital simulator was beyond the scope of the present study. Such validation will be required before practical field implementation to quantify hardware-dependent processing delays and overall system response time.

## Conclusion

This paper presented an intelligent protection framework for fault detection, classification, and location in the IEEE 16-bus distribution system through the integration of Discrete Wavelet Transform (DWT), Support Vector Machines (SVMs), and cloud-assisted IoT monitoring. In addition to fault analysis, the proposed framework was evaluated under various non-fault operating conditions, including overload events, load switching, and capacitor switching operations, thereby enhancing its capability to distinguish actual faults from other network disturbances. This capability reduces the likelihood of false fault indications and improves the reliability of protection decision-making under dynamic operating conditions. Within the investigated simulation scenarios, the proposed methodology achieved 100% fault classification accuracy and fault-location accuracies ranging from 97.95% to 99.88%. Furthermore, the proposed framework maintained high performance under renewable-integrated operating conditions, confirming its effectiveness in active distribution networks with distributed generation penetration.

A comprehensive evaluation of wavelet decomposition levels and mother wavelets revealed that the eighth decomposition level provided the most informative fault-related features, while the Sym3 mother wavelet offered the most favorable balance between prediction accuracy, computational efficiency, and training time. Among the investigated machine-learning models, Cubic SVM exhibited superior fault classification performance, whereas Linear SVM achieved the lowest RMSE values for fault location, making it the most suitable choice for the location task both with and without distributed generation. The obtained results demonstrate the effectiveness of combining DWT-based feature extraction with SVM-based decision-making for intelligent distribution system protection. The scientific contribution of this work lies in the development and systematic validation of an integrated intelligent protection framework that combines optimized DWT-based feature engineering, SVM-based intelligent decision-making, and cloud-assisted monitoring within a unified protection architecture, rather than in the introduction of new signal-processing or machine-learning algorithms. Furthermore, the integration of cloud-assisted monitoring with the proposed local intelligent protection framework enhances situational awareness and supports data-driven monitoring and management of modern power distribution systems while maintaining complete independence from the real-time protection decision path.

Nevertheless, the reported performance is limited to the investigated simulation environment and operating conditions. Additional validation using noisy measurements, hardware-in-the-loop platforms, embedded hardware implementations, and experimentally acquired field data is required to comprehensively assess the robustness, scalability, and practical applicability of the proposed framework in real-world distribution networks. Moreover, although the implemented ThingSpeak platform successfully demonstrated cloud-assisted monitoring and remote accessibility of fault-related information, practical deployment of IoT-enabled protection systems requires further investigation of communication latency, cybersecurity requirements, synchronization mechanisms, communication reliability, and edge–cloud coordination strategies. Together, these research directions represent the logical next stage toward comprehensive validation, practical deployment, and real-world implementation of the proposed intelligent protection framework in modern active distribution networks.

## Data Availability

The data that support the findings of this study are available from the corresponding authors upon reasonable request.
